# Narrative review on century of respiratory pandemics from Spanish flu to COVID-19 and impact of nanotechnology on COVID-19 diagnosis and immune system boosting

**DOI:** 10.1186/s12985-022-01902-2

**Published:** 2022-10-24

**Authors:** Walid F. Elkhatib, Shereen S. Abdelkareem, Wafaa S. Khalaf, Mona I. Shahin, Dounia Elfadil, Alaa Alhazmi, Ahmed I. El-Batal, Gharieb S. El-Sayyad

**Affiliations:** 1 Microbiology and Immunology Department, Faculty of Pharmacy, Ain Shams University, African Union Organization St., Abbassia, Cairo, 11566 Egypt; 2 Department of Microbiology and Immunology, Faculty of Pharmacy, Galala University, New Galala City, Suez, Egypt; 3 Department of Alumni, School of Pharmacy and Pharmaceutical Industries, Badr University in Cairo (BUC), Entertainment Area, Badr City, Cairo, Egypt; 4 Department of Microbiology and Immunology, Faculty of Pharmacy (Girls), Al-Azhar University, Nasr City, Cairo, 11751 Egypt; 5 Zoology Department, Faculty of Tymaa, Tabuk University, Tymaa, 71491 Kingdom of Saudi Arabia; 6 Biology and Chemistry Department, Hassan II University of Casablanca, Casablanca, Morocco; 7 Medical Laboratory Technology Department, Jazan University, Jazan, Saudi Arabia; 8 SMIRES for Consultation in Specialized Medical Laboratories, Jazan University, Jazan, Saudi Arabia; 9 Drug Microbiology Laboratory, Drug Radiation Research Department, National Center for Radiation Research and Technology (NCRRT), Egyptian Atomic Energy Authority (EAEA), Cairo, Egypt

**Keywords:** SARS-CoV-2, Spanish flu, Nanotechnology, Immune system, Respiratory pandemics

## Abstract

The rise of the highly lethal severe acute respiratory syndrome-2 (SARS-2) as corona virus 2019 (COVID-19) reminded us of the history of other pandemics that happened in the last century (Spanish flu) and stayed in the current century, which include Severe-Acute-Respiratory-Syndrome (SARS), Middle-East-Respiratory-Syndrome (MERS), Corona Virus 2019 (COVID-19). We review in this report the newest findings and data on the origin of pandemic respiratory viral diseases, reservoirs, and transmission modes. We analyzed viral adaption needed for host switch and determinants of pathogenicity, causative factors of pandemic viruses, and symptoms and clinical manifestations. After that, we concluded the host factors associated with pandemics morbidity and mortality (immune responses and immunopathology, ages, and effect of pandemics on pregnancy). Additionally, we focused on the burdens of COVID-19, non-pharmaceutical interventions (quarantine, mass gatherings, facemasks, and hygiene), and medical interventions (antiviral therapies and vaccines). Finally, we investigated the nanotechnology between COVID-19 analysis and immune system boosting (Nanoparticles (NPs), antimicrobial NPs as antivirals and immune cytokines). This review presents insights about using nanomaterials to treat COVID-19, improve the bioavailability of the abused drugs, diminish their toxicity, and improve their performance.

**Graphical Abstract:**

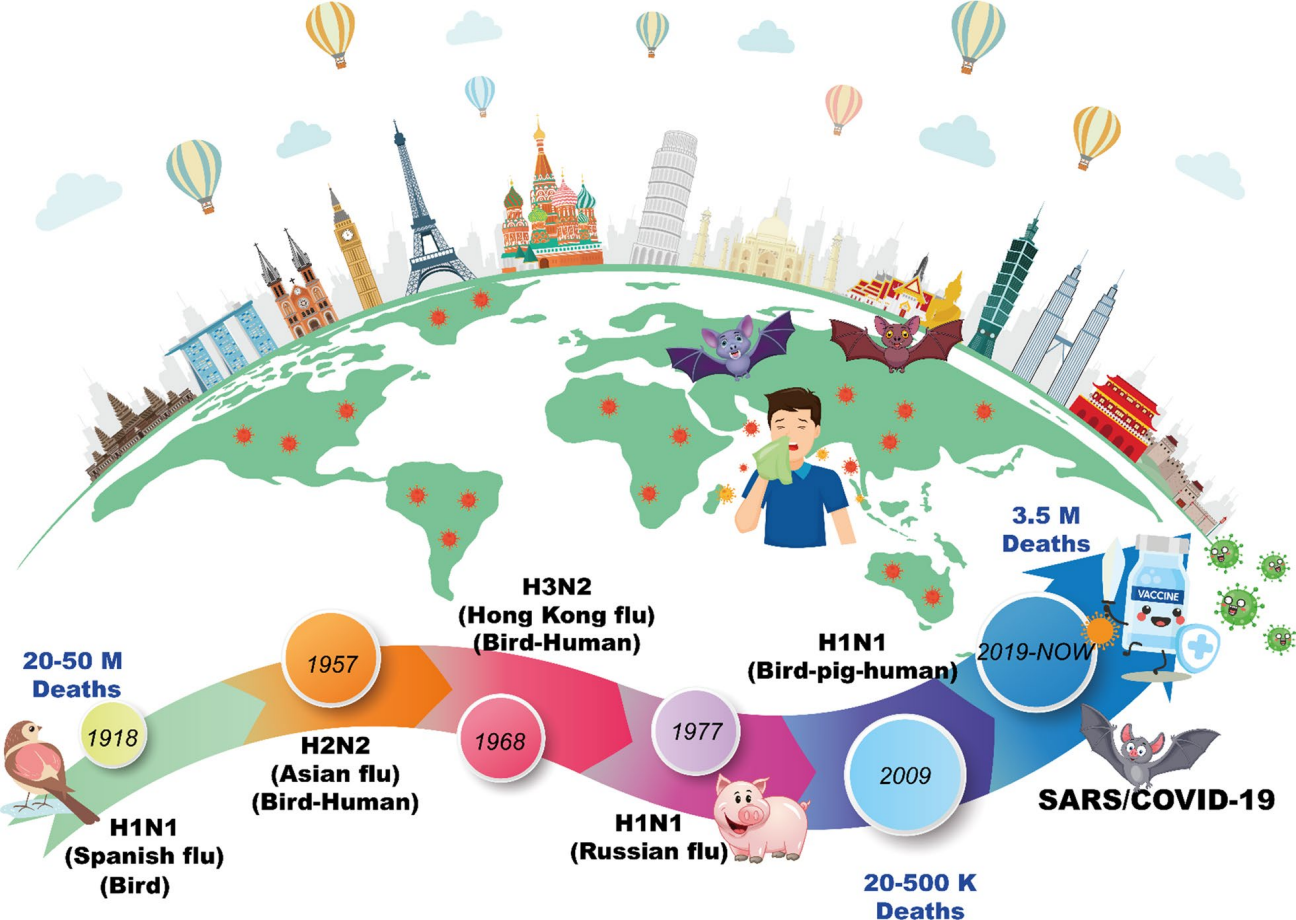

## Introduction

The rapidly spreading throughout the existing highly contagious Severe-Acute-Respiratory-Syndrome-2 (SARS-2) or so-called Coronavirus 2019 (COVID-19) disease, reminded us of other pandemics that happened in the last century (H1N1 Spanish flu) and continued in the current century by (SARS, MERS, and COVID-19) [1–3]. After a series of respiratory viral pandemic diseases that started in 1918–1919 by a mysterious and fatal disease called H1N1 Spanish Flu and some researchers called it Mother of Pandemics [4], due to this pandemic has infected more than a third of the world's population and claimed an approximate 50 million lives, with untypically extreme clinical symptoms in young, formerly disease-free adults, the pandemic has been a major cause of death [5]. In this regard, SARS-CoV-2 and 1918 influenza A/H1N1 viruses have some common properties, such as being of similar basic reproduction numbers (R0), varying from 2 to 4, and similar shedding patterns from infectious patients, and hence likely to have similar generation gaps. In tandem, COVID-19 may have a latency period similar to that of influenza [6]. Then, in the current millennium, the world has witnessed persistent viral attacks from a novel viral family called Coronaviruses (CoVs) [7]. CoVs, containing an Orthocoronavirus subfamily and a Torovirinae subfamily. The Orthocoronavirinae subfamily comprises four genera: the alpha coronavirus, the beta coronavirus, the gamma coronavirus and the delta coronavirus [8]. Beta coronavirus genera encompass from Severe-Acute-Respiratory-Syndrome (SARS), Middle-East-Respiratory-Syndrome (MERS), human CoV-229E (HCoV-229E), HCoV-OC43, and Corona Virus-2019 (COVID-19) [3].

In 2002, 2012 and 2019, the world was attacked by three viral respiratory diseases by the SARS, the MERS and the COVID-19, accordingly. Corona viruses are enveloped, non-segmented, positive-sense, monostranded RNA viruses that show a characteristic appearance under negative-staining electron microscopy [9].

The WHO reported that in the southern Chinese province of Guangdong, on November 2002, no update was received from the Government of China up to the month's end of March, a massive 792 cases and 31 deaths were reported. The ministry of health of China reported more than 8,000 cases.It is estimated that there were 1 thousand cases of disease and about 774 deaths, giving a lethality rate of about 7%. The reservoir host of the infection is thought to be the Asian civet cat (*Paguma larvata*). It was expected that the host-to-human transmission sites would be open markets, as is the case with the current COVID-19 outbreak. [10]. The global SARS epidemic was successfully controlled in July 2003, and no cases of SARS have been reported since 2004. [11]. The emergence of SARS was followed by MERS as the second most important coronavirus causing a world-wide public health emergency. First appeared in Saudi Arabia (KSA) in 2012 when a patient aged 60 with severe pneumonia [10]. An epidemic of the virus only became apparent in 2014, with a total identified case count of 662 and a case fatality index of 32.97%. From 2014 to 2016, 1364 cases were observed in KSA [10]. Overall, 27 countries have been affected by MERS during the epidemics, in Asia, Europe, the Middle East, and North America. [12]. The cases identified beyond the Middle East, including the South Korea (SK) epidemic in where 186 people have been found to be infected as a result of a supraspreading, have involved transplant recipients who have already been infected in the Middle East. Since 2012, a total of 2494 lab-confirmed cases of MERS have been reported, with 858 associated deaths (case-fatality ratio of 34.4%) [10].

With regard to COVID-19, WHO has raised the risk level of the CoV crisis to "very high" on 28 February 2020. On 11 March, as COVID-19 incidents outside China have increased by a factor of 13 and the number of infected countries has increased threefold to over 118,000 registered cases in 114 different countries, with more than 4,000 fatalities, the WHO declared COVID-19 a global pandemic. Governments around the world are working to put countermeasures in place to hold the potentially deleterious effects. Health organizations are coordinating the flow of information and issuing directives to best minimize the impact of the pandemic threat. Meanwhile, researchers from all over the world are working intensively and information on the transmission mechanisms, the clinical spectrum of the new diagnosis of the disease, prevention and treatment approaches is developing rapidly. Many unknown factors concerning the virus-host dynamic and the progress of the epidemic remain, including the timing of its peak [13].

Nanomaterials (NMs) have specific features that are unique, which characterize them as outstanding materials able to apply in spectrum devices, sensors, and techniques used in virus’s detection, treatment, and virus’s elimination from the environment [14]. A major part of the applications of NMs is to predict and treat viruses in the health care and environment. In this review, we are presenting a brief about some of these uses.

## Origin of pandemic respiratory viral diseases

The avian influenza is distinguished for its capacity to contaminate various animal diversity, including species such as bats, birds and mammals. Even though successful interspecies transmission is rare, it plays a pivotal role in the generation of new vectors of the pandemic [14]. In the pandemic of 1918–1919, March 1918 was the start of the spring wave. It spread across the Europe, US, and Asia over the next six months. Although levels of disease were high, mortality rates in most places were not significantly higher than usual. A second or autumn wave spread around the world from September to November 1918 and was very fatal. In many countries, a third wave occurred in early 1919. Contemporary observers concluded from the clinical similarities that they were seeing the same infection disease in successive waves [4]. The mild forms of the epidemics in all three waves were typical of the influenza seen in the 1889 pandemic and the avian flu epidemic and previous inter-pandemic years. In view of this, even the quick progression from uncomplicated influenza infection to fatal pneumonia, characteristic of the autumn and winter waves of 1918–1919, was observed in the few severe cases in the spring wave [4, 15].

Up until 2003, only 2 CoVs, Human CoV 229E (HCoV-229E) and HCoV-OC43 have been known to lead to human disease [16]. It manifests as mild symptoms such as a common cold in adults and more serious illness in infants, the old and immunocompromised people. In November 2002, numerous exceptional cases of "*atypical pneumonia*" of unexplained reason reported in the city of Foshan, Guangdong Province, In China, where many health staff have been contaminated [17]. This was introduced to Hong Kong on 21 February 2003 by a doctor who dealt with similar cases of SARS in the Chinese mainland, which resulted in widespread of serious pneumonia in Hong Kong and labelled by WHO as “severe-acute respiratory-syndrome” on March 15, 2003 [18, 19]. Months passed, and a number of incidents of SARS have been identified prior to SARS-CoV was identified. A new b-CoV (SARS-CoV) of lineage B was confirmed as the cause of the SARS pneumonia cases on 22 March 2003. The SARS-CoV pandemic has spread to 29 countries and regions. It was clear that the world's health, medical and scientific development communities were not sufficiently prepared for the emergence of SARS. [18]. Human-to-human chains of transmission have emerged in Canada, Toronto, Chinese Taipei, Hong Kong, China, Vietnam Singapore and Hanoi. The SARS epidemic had a short history and the WHO announced the winding down of the SARS epidemic in July 2003 [18].

Ten years since the last sign of SARS-CoV, in June 2012, a man died in KSA of serious pneumonia and kidney weakness [20]. A newly discovered corona-virus, the Middle-East-Respiratory-Syndrome-Coronavirus (MERS-CoV), has been identified from his sputum [21]. A group of severe-respiratory illness cases had emerged in April 2012 in Jordan in a hospital and were diagnosed in retrospect as MERS12, and a group of three MERS infected cases in the UK were detected in September 2012 MERS-CoV has continued to rise and expand outside the "Arabic peninsula”. As a consequence of travel by contaminated individuals; frequently these newly transmitted MERS cases have resulted in hospital-acquired transmission.[22]. In May 2015, the MERS outbreak in SK was triggered by a single returnee from the Middle East and affected sixteen clinics and 186 cases. As of 26 April 2016, 1,728 MERS cases have been confirmed, of which 624 fatalities in 27 different countries [22].

**Fig. 1:**
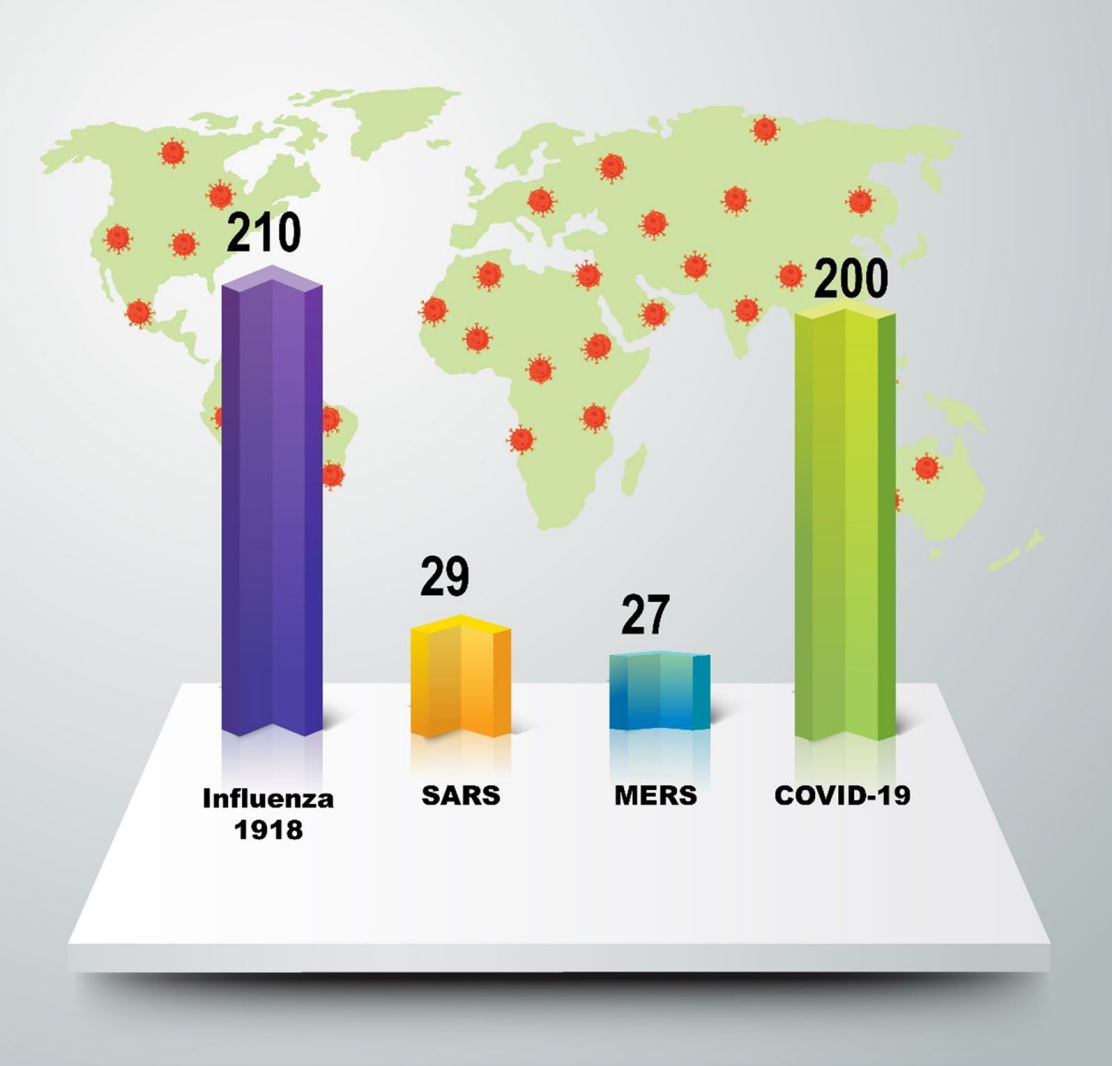
Illustration of some essential information about the number of countries affected by respiratory viral pandemics from 1918 until 2022

A pneumonia group of cases linked to a recently discovered β-coronavirus appeared in Wuhan, China, in December 2019. On January 12, 2020, the World Health Organization (WHO) designated this coronavirus as the 2019-novel coronavirus (2019-nCoV) (WHO). International Committee proposed naming the newly identified coronavirus as SARS-CoV-2, both reported on 11 February 2020. Chinese scientists rapidly isolated SARS-CoV-2 from a patient on 7 January 2020. They came out to genome sequencing of the SARS-CoV-2. As of 1 July 2021, 91,833 cases of COVID-19 have been confirmed in mainland China including 4636 deaths. The basic reproduction number “R0” of SARS-CoV-2 has been assessed by studies to be around 2.2 or more (range 1.4–6.5), and family pneumonia outbreak groupings add to the proof of a steadily growing COVID-19 epidemic through human-to-human transmission (Fig. 1) [23].

According to the WHO (https://covid19.who.int/); globally, as of 8:36 pm CEST, 14 April 2022, there have been 500,186,525 confirmed cases of COVID-19, including 6,190,349 deaths, reported to WHO. As of 18 April 2022, a total of 11,307,908,653 vaccine doses have been administered.

## Reservoir and mode of transmission

**Fig. 2:**
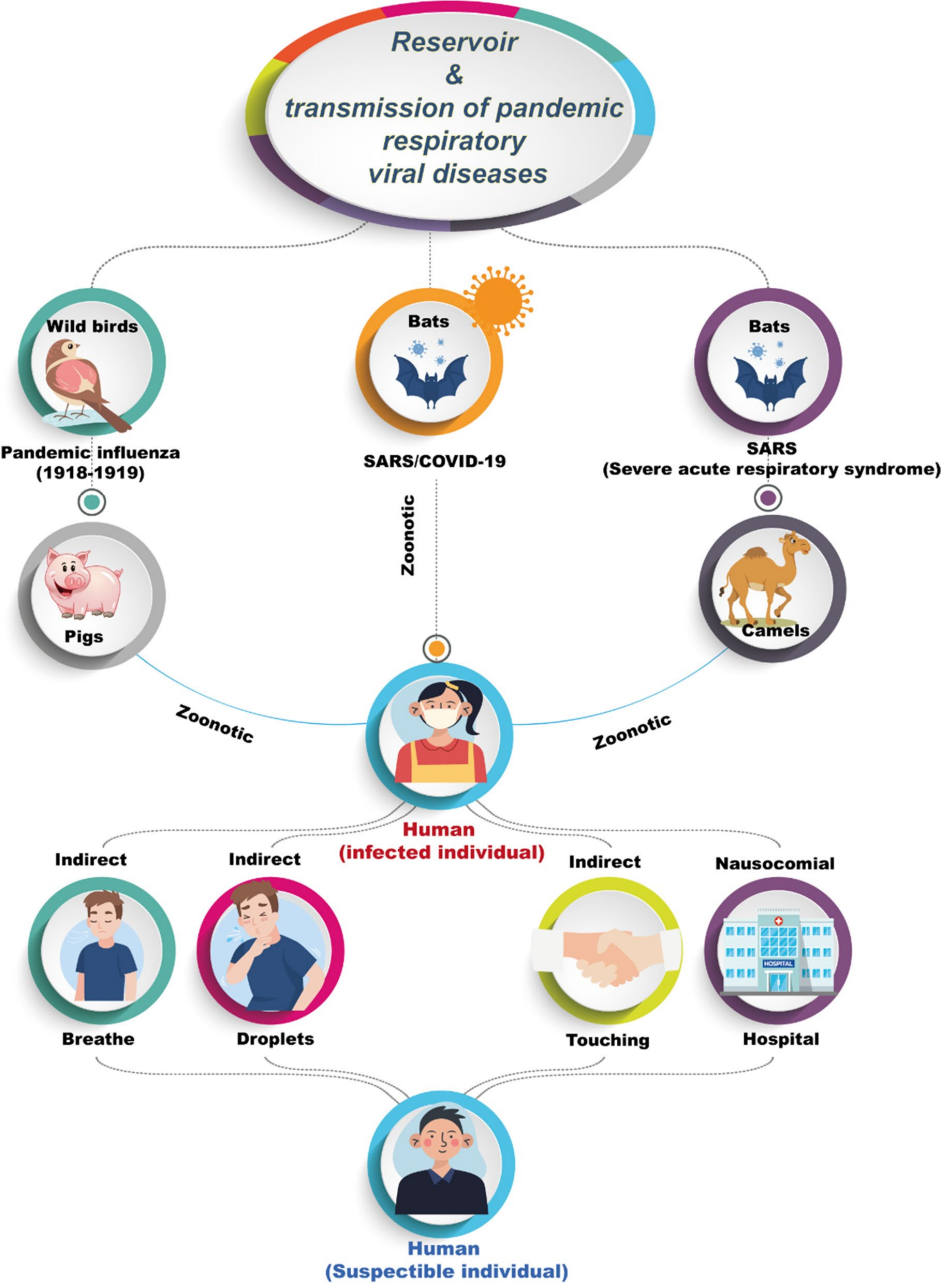
Illustration of the reservoir of pandemic influenza viruses in 1918. Wild aquatic birds and other species are considered to be a source of all influenza viruses. Even though Transmission between humans and pigs has already been demonstrated and confirmed, there has been a less direct transmission of avian to humans Frequent (such as those with subtypes H9N2 and H5N1) but often fatal. Regarding Beta coronavirus, Bats are the reservoir of a wide variety of coronaviruses, including severe acute respiratory syndrome coronavirus (SARS-CoV)-like viruses in 2002–2003, Middle East Respiratory Syndrome (MERS) in 2012 besides to camels as the natural host of MERS-CoV and COVID-19 in 2019–2020

Influenza viruses in different species are known to originate in wild waterfowl. (Fig. 2) [24]. Whilst human-pig transmission subtypes have already been shown and substantiated, direct transmission between birds and humans has been less prevalent (as in the case of H9N2 and H5N1 subtypes) but has in some cases resulted in fatalities. [25].

Regarding the reservoir of coronaviridae family which consists of beta-coronavirus that include three pandemics recently (SARS, MERS, COVID-19). Bats are a huge natural reservoir wide range of CoVs, including SARS-CoV-like and MERS-CoV-like viruses (Fig. 2). Following the virus's genomic sequence, COVID-19 was studied, the genome for Bat CoV RaTG13 showed 96.2 percent overall identity of the genome series, indicating the CoV bat Even human SARS-CoV-2 may have the same parentage. In contrast, bats aren't accessible for purchase in this seafood marketplace business. By the way, synchronization of protein sequences and further phylogenetic study revealed similar residues receiver was found in several species, which offered more possibilities for alternate intermediate hosts; For starters, tortoises, pangolin, and snacks [23].

All pandemics respiratory viruses are zoonotic airborne RNA viruses that are rarely transmitted between native forms of humans but could mutate to make human transmission more effective. Frequent and approved transmission routes droppings (> 5 mm diameter, flying Transmission' < 1 m) make contact with the nose with viable viruses, mouth, eyes, or upper airway and 'airborne transmission' where droplets (5 mm diameter) are kernels [27]. 2002 and 2003 were examples of the H1N1 influenza pandemic in SARS and 2009.

The function of 'direct communication of touch' (without the possibility of polluted surfaces) and 'indirect touch propagation' (including infected surfaces) of the distribution of such pandemic potential viruses have been controversial. Nonetheless, various reports and investigations have reported that indirect communication transmission is prevalent. The transmission path for other respiratory viruses, as well as influenza, under certain conditions [26].

## Replication of SARS-CoV-2 virus

Influenza virus replication occurs at the cellular level mainly in the epithelial cells of the intestinal tract in birds and in the epithelial cells of the respiratory tract in humans and other mammals [27]. In humans, ribonucleo-proteins (vRNPs) are subsequently transmitted into the nucleus of the diseased cells, in which viral RNA transcripts and replicates through the enzymatic activity of the viral polymerase complex attached to vRNPs [28]. The replication of viral RNA occurs via a positive intermediate, the complementary ribonucleoprotein complex [29]. Transcription of viral-RNA produces positive-stranded mRNA that is cap-linked and polyadenylated and then exported to the cytoplasm to be translated into viral proteins. [30]. Virus newly synthesised polymerases (PA, PB1,and PB2) and viral NP are imported into the nucleus to increase the rate of viral-RNA synthesis, while the viral membrane proteins HA, NA and M2 are transported and incorporated in the plasma membrane [31].

MERS-CoV and SARS-CoV have a specific coding mechanism in which about two thirds of the “viral RNA” is translated into two giant poly-proteins, while the remaining viral genome is transcribed in one nested series of subgenomic mRNAs [22]. Both pp1a and pp1ab, polyproteins encode sixteen non-structural proteins. (nsp1–nsp16) which bring up the viral replicase transcriptase complex [32]. The polyproteins are cleaved by papain-like protease (PLpro; corresponding to nsp3) two proteases, and The protease, 3C-like protease (3CLpro; corresponding to nsp5). nsps re-arrange membranes derivated from the rough endoplasmic reticulum (RER) into dual-membrane vesicles, in which the virus transcription and replication take place. The exoribonuclease (ExoN) function of nsp14 is a unique feature of coronaviruses, which supplies the correction capacity necessary to sustain a largescale RNA genome without accumulating harmful mutations. MERS-CoV and SARS-CoV transcribe 9 and 12 subgenomic RNAs respectively. These encode the four structural proteins, namely the spike protein (S), envelope (E), nucleocapsid (N),,membrane (M) and several accessory proteins that do not participate in viral replication but interfere with the host's innate immune response or whose function is unknown or misunderstood.

The envelope “E” spike glycoprotein “S” clings to its cellular receptor, angiotensin converting enzyme 2 (ACE2) for SARS-CoV and dipeptidyl peptidase 4 (DPP4) for MERS-CoV. The “viral RNA genome” is delivered into the cytoplasm after membrane fusion, emissions to the host cell membrane or to the endosome membrane. The RNA is unwrapped to permit translation of the two polyproteins, transcription of the subgenomic RNAs and replication of the viral genome. The resulting envelope glycoproteins are introduced into the RER or Golgi membranes; genomic RNA and nucleocapsid proteins coming together to form the nucleocapsids. Virus particles bud in the ER-Golgi intermediate compartment (ERGIC). The virus-containing vesicles then fuse with the plasma membrane to deliver the virus [22].

**Fig. 3:**
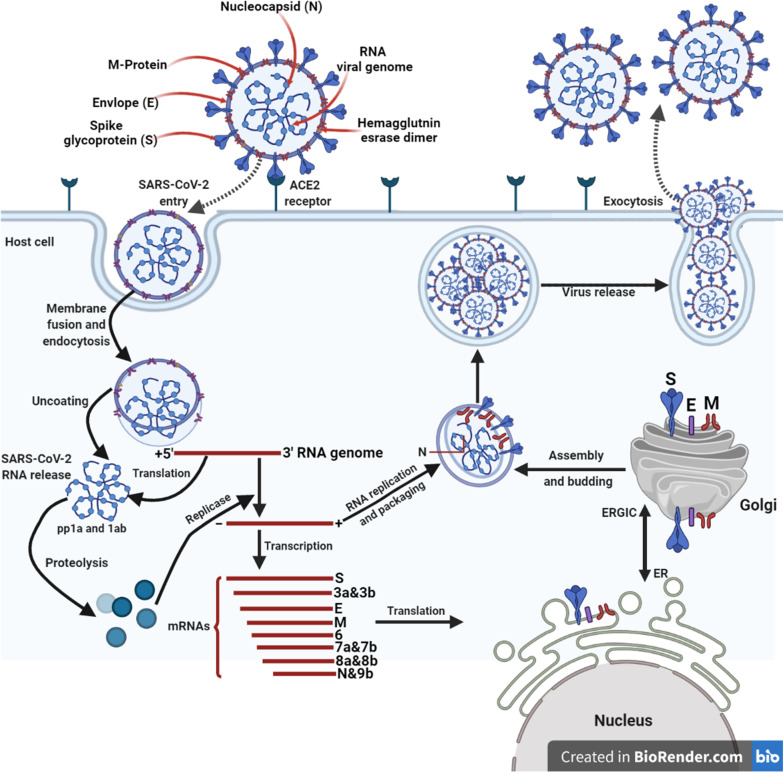
The replication of of respiratory SARS-CoV-2 virus inside the host cell

Regarding COVID-19 (Fig. 3), genomic RNA is utilized as a scaffolding to directly translate polyprotein 1a/1ab (pp1a/pp1ab), which encodes non-structural proteins (nsps) to make the replication-transcription complex (RTC) in double membrane vesicles (DMV). Eventually, a nested set of subgenomic RNAs (sgRNAs) is synthesised by the RTC in a discontinuous mode of transcription. These subgenomic messenger RNAs (sgRNAs) have common 5′-leader and 3′-terminal sequences. and Subsequent acquisition and Transcription termination of a leader RNA occurs at transcriptional regulatory sequences situated between open reading frames (ORFs). These minus-stranded gRNAs act as a template for subgenomic mRNA production.

A typical CoV genome and subgenomes include at minimum six ORFs. The first"ORFs (ORF1a/b), which account for about two-thirds of the total genome length, code for 16 nsps (nsp1-16), with the exception of Gammacorona virus, which has no nsp1. There is a − 1 frame shift between ORF1a and ORF1b, resulting in the production of two polypeptides: pp1a and pp1ab. These polypeptides are treated into 16 nsps by the virus-encoded chymotrypsin-like protease (3CLpro) or master protease (Mpro) and one or two papain-like proteases. Other ORFs on the third of the genome near the 3′-tip encode at least four major structural" proteins: (S), (M), (E) and (N) [33]. Various CoVs encode specific structural and accessory proteins,like the HE protein, the 3a/b protein and the 4a/b protein, in addition to these four basic structural proteins. CoV sgRNAs are used to translate all structural and accessory proteins [34].

## Viral adaption needed for host switch and determinants of pathogenicity

Influenza A virus will turn hosts and create newly developed lineage [35]. This infection, known as zoonotic offers a chance to adapt the virus to the next host, and the resulting pandemics. When influenza A virus enters the body, the grippe HA (hemagglutinin) molecule accepts sialic acid (N-acetylneuraminic) around the top of the host cell [36]. HA is a transmembrane type of glycoprotein as homotrimer introduced to the virus sheet. Every monomer is composed of two subunits, HA2 and HA1. In the endosome low pH region, cleaved HA with a fusogenic HA2 stalk domain fusion mediates of the endosomal membrane with the viral membrane, which makes viral entry strong ribonucleoprotein (vRNP) to host cell [37].

The"vRNP complex comprises of 8 single-stranded, negative-sense nucleoprotein (NP) vRNA, and RNAs for influenza-A Polymerase"(PA, PB1, and PB2 compound)[28]. With subsequent fusion, the vRNP complex can be liberated in the cell's cytoplasm, after which it enters the nucleus by successful conveyance [37]. The nucleus is where the RNA synthesis of all influenza viruses takes place. Begin transcription process; RNA polymerase virus binds to highly retained and almost complementary 13 at the end of the 5' nuclear power and 12 at the end of the 3' nucleotide"eight segments. Nevertheless, the polymerase influenza virus has no inherent capping activity. It summarizes RNAs use 5 'host cap pre-mRNAs viral massager A special "cap snatching" mechanism mediation PB1 and PB2 derived from cellular transcriptions protein [28]. Nonstructural NS1 protein affects viral morphogenesis later in the viral replication cycle particles. However, they are not viral structural particles [38].

Receptor identification represents the initial stage of viral infection of host cells and one of the most essential factors in viral infection and pathogenesis [39]. While many other host- and virus-related factors can also influence the efficiency of infection and replication of the virus in a specific host, these factors only come into play once the virus is linked to a cell membrane receiver [40]. Coronaviruses (CoVs) have an enveloped, single-stranded-RNA genome that encodes four membrane proteins, namely spike, membrane, envelope, and nucleocapsid proteins as shown in Fig. 3 [2, 41]. S proteins are important for a viral entry concerning pathogenicity [42]. On the SARS-CoV envelope “E”, a trimeric S mediates the penetration of the virus into host cells. It first links to its host receiver, the angiotensin 2 converting enzyme (ACE2), and fuses the host and virus membranes afterward [43]. A given receptor-binding-domain (RBD) on the “S” SARS-CoV is sufficient to bind with high-affinity to ACE2 [44]. An important element in the pathogenesis of SARS-CoV and cross-species infections has been identified as the RBD/ACE2 binding affinity [45]. The experimental cross-reactivity of anti-SARS-CoV antibodies with 2019-nCoV spike proteins, that might have a significant consequences for the rapid manufacturing of antibodies and vaccines to combat 2019-nCoV, is therefore urgently needed [46].

## Virulence factors of pandemic viruses

Virulence factors are considered one of the vital element which plays a prominent role in virus adaption into the host cell [47]. Regarding pandemic influenza, Haemagglutinin (HA) is part of the surface glycoprotein of the virus. with two main roles in the very earliest phase of virus replication: membrane fusion and receptor linking [48]. HAs of high Avian influenza virus pathogen set an important contribution in virulence. They usually have a specific sequence (i.e. a sequence of basic amino acids at the cleavage site that helps to the prevalence of pathogenicity). [49]. This pattern, although it is not reflected in the HA sequence of 1918. Nevertheless, it had been shown that a reasserting virus with the genetic history 1918 HA repeated at a significantly elevated titer in the lungs, and with a large influx of lungs from neutrophils and alveoli macrophages caused severe pulmonary damage. The real 1918 virus with significant morbidity and subsequent death showed similar results. These results indicate an important role in the disease of the 1918 virus for the HA gene [50]. The high-virulence area (s) of HA has not yet been discovered. The other central factor in the 1918 outbreak is virulence. One unusual characteristic of the pandemic of 1918 was that many people have passed away of viral pneumonia; viral flu viruses in the pulmonary system of infected persons usually replicate poorly and often result in life-threatening viral pneumonia [51]. In the pulmonary system of infected filaments and non-human primates, we record an effective replication of the 1918 virus, which contributed to viral pneumonia.

In comparison, the lungs of infected animals did not have a contemporary human H1N1 virus, even though it reproduced in the nasal cavities. Therefore, we conclude that the 1918 virus's capacity to expand in the lungs is related to its high human virulence [51]. It is also noted that high virus titers were based on the NP genes and polymerase in the lungs of infected ferrets in 1918 [52]. Polymerase genes also are significant in the pathogenicity and transmission of the mouse in ferrets [53]. These results strongly involve viral RNA polymerase complex in the successful transmission of the virus to the low respiratory tract and indicate that, in combination with a particular HA, it may be sufficient to induce fatal pneumonia during the pandemic of 1918–1919 [54]. Pathogenicity can also be correlated with other viral factors like the case of pandemic NA, NS1, PB1-F2 and others in 1918. The pro-apoptotic viral protein PB1-F2 needs only the shift in one amino acid at the 66th position to enhance the virulence of the virus in 1918 [51]. The 1918 PB1-F2 expression encourages pulmonary pathology in primary viral and secondary bacterial infections [51].

Respecting beta coronaviruses (SARS, MERS, and COVID-19), they contain E protein consisting of several active motifs between 76 and 109 CoV-dependent amino acids given its limited size [55]. Modification or suppression of E protein in different CoVs resulted in viruses with different phenotypes and unusual interrelationships between the virus and the host including stress induction and protein reactions or changes in concentrations with cellular ion because of E protein ion channel activity [55, 56]. All these practices have an important effect on the pathogenesis of CoV. Furthermore, in COVID-19 “S” is the main defining of cell tropism and therefore interspecies transmission of CoVs, since it binds the virus to a cellular receptor and then catalyzes membrane fusion entry of the virus [57]. The electron-microscopy 3D structure of the 2019-nCoV viral S showed its similarity with the S of the other COVs [57]. The further characteristics of other CoVs may thus be deducted. Viral S is a transmembrane type I transmembrane protein with an n-terminal cleavable signal peptide, a large and highly n-glycosylated e, a transmembrane, and cytoplasmic tail embedded in an S-cyllated residue cluster [57]. The ectodomain has been divided into the highly variable S1 domain between the genera and the S2 domain that is more conserved and catalyzes membrane fusion. The recipient-binding operation causes pathogenicity [58].

## Symptoms and clinical manifestations

**Fig. 4:**
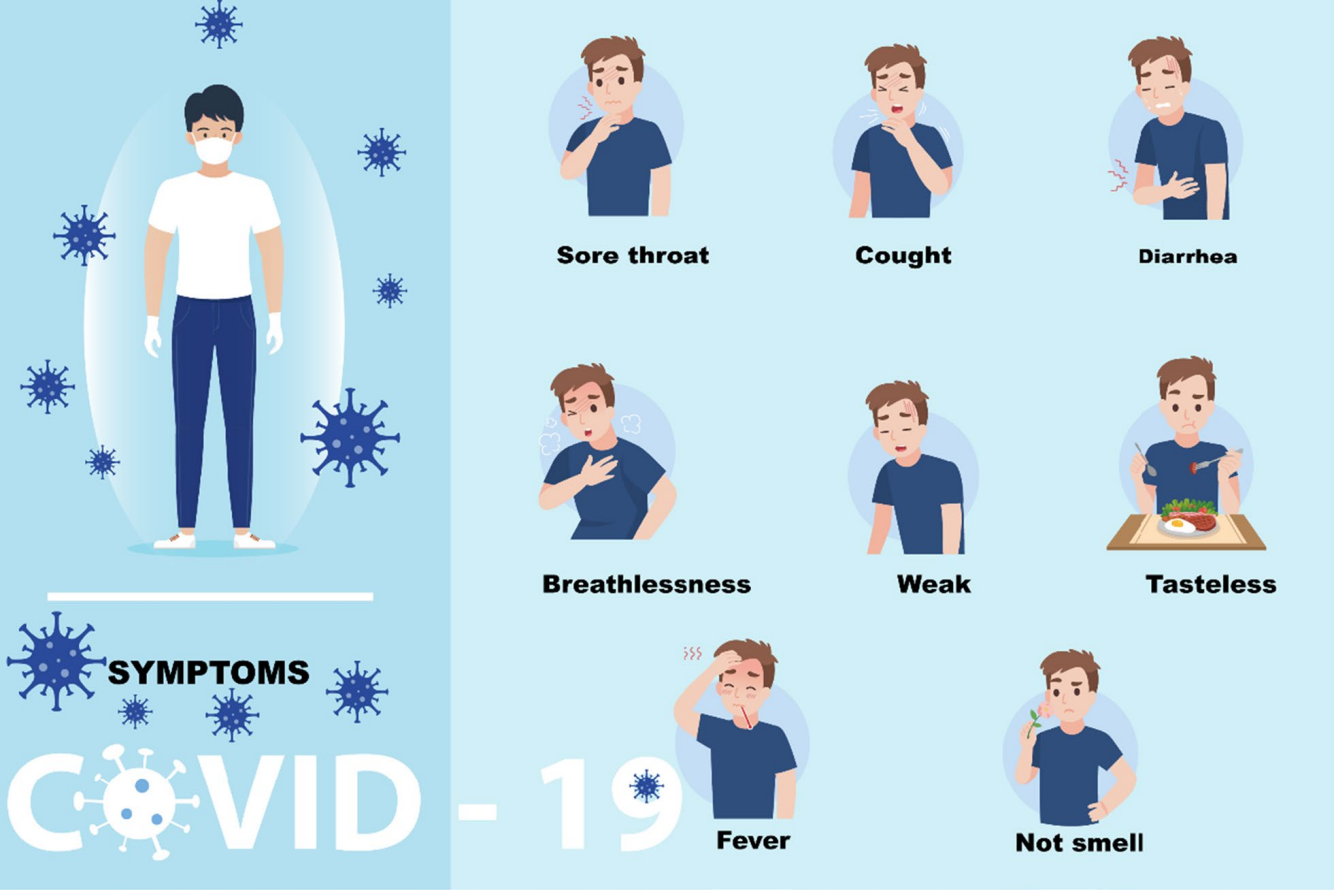
Illustration of some essential information about the most common symptoms among individuals who affected by any pandemic viral respiratory disease (H1N1 influenza, SARS, MERS, and COVID-19)

The pandemic H1N1 influenza virus in 1918–1919 witness variations in signs and symptoms according to many factors such as the severity of the cases, individual's age, and season [59]. The mild, unclear illness as predominate in the spring herald waves included symptoms of the upper respiratory tract, such as sore throat, nasopharyngitis, and cough, as well as systemic manifestations of fever, myalgia, and prostration (Fig. 4) [60]. Epistaxis has been carried out in both mild and severe cases [61]. The physician reporting Three thousand cases at Camp Fremont noted that epistaxis was a common characteristic of the entire pandemic [60]. This was considered a characteristic of the disease as blood always poured from the nose and mouth of the patient. The duration of moderate illness was generally limited to 72 h. Typically, the cough was not productive. Fever was prevalent up to 104° F. Sometimes sudden and extreme prostration [62]. One definition of the patient as "quickly or almost unexpectedly seized with a sense of prostration that was completely incapable of doing what he could. There was significant respiratory distress in patients with severe illness [62]. Their symptoms included remarkably intense cyanosis, hunger in the air, reduced awareness, and diffuse bubbling rales of highly progressive lung edema (Fig. 4). The cyanosis of heliotropic cyanosis found in some patients before death after the heliotrope flower's deep blue or purple color. Physicians also first note that the lips and ears are intensely blue before focusing on the rest of the face [31]. In a letter to a colleague's doctor, some described the color as purplish-black and one Scottish doctor who works at camp Deven noted that "the men color to white are not easy to distinguish." In the abnormal pigment, a doctor determined that cyanosis is due to extensive exudations in the alveoli preventing proper oxygenation when a repeated spectrographic control of the patient's blood was not found [60]. Two psychiatric conditions related, acute respiratory disturbance. Rapid mortality syndrome (ARDS), and fatal cases were identified of bronchopneumonia. A secondary bacterial infection leading to bronchopneumonia caused the most deaths with pneumonia, except for those killed in 1918 after the epidemic of H1N1 [60]. Initial leukopenia was followed by bronchopneumonia leukocytosis [63]. Brundage and Shanks [64], recorded a median period of 7–10 nights from the onset of illness and several deaths > 15 days after the onset, in conjunction with secondary bacterial pneumonia, for the most affected population.

In the case of SARS, Fever, chilling, rigors, myalgia, dry toxins, dyspnea, malaise, and headache are the main distinguishing clinical features of SRAS [65]. More popular are sore throats, diarrhea, rhinorrhea, nausea, vomiting, and swelling [65]. In 40–70% of SARS patients, watery diarrhea was present. It tended to happen about 7 days after the onset of the disease [18]. Two patients complicated with epileptic status was detected in serum and cerebrospinal fluids. Elderly SARS-CoV infected patients may develop a low appetite, decrease in overall well-being, fall-fracture, and uncertainty, but may not be able to mount febrile responses to some elderly people [66].

In comparison, SARS-CoV infection was usually mild in kids under the age of 12. In contrast, infection in adult children was close to that in adults [67]. SARS-CoV-ac-Quired infection was connected to a lethality rate of 25%. during pregnancy, a high incident of spontaneous abortion, preterm delivery, and delayed development of the intrauterine child without perinatal SARS-CoV infection [18].

Adults who become infected with MERS-CoV may develop a range of illness and disease severity, from asymptomatic to slight, moderate or severe (Fig. 4) [68]. The time of incubation is from 2 to 14 days. Low-grade fever, runny nose, sore throat, dry cough and myalgia can occur in patients with mild infections. Patients with serious infections have acute pneumonia Syndrome of respiratory pain, a multilateral scheme organ failure, and disease. Furthermore, fellow members measure pneumonia progression by scoring the number of chest x-ray lung zones in patients with severe infection, showing abrupt progression after approximately seven days and severity pneumonia. The symptoms peaked afterward about fourteen days. MERS-CoV is greater in the lower respiratory tract samples than in the upper respiratory tract samples. Also, extrapulmonary characteristics are common, including myalgia. Back to half of all MERS-CoV patients, a third of critically ill people, including abdominal pains, nausea, vomiting, and diarrhea, are experiencing acute kidney injury. A third are gastrointestinal, and MERS-CoV in stool can be found [68].

Regarding COVID-19 fatigue and cough is myalgia or tiredness [69], most frequently reported symptoms. Expectoration, headache, haemoptysis and diarrhoea less frequent symptoms [70, 71], and in over half the patients, dyspnea developed (Fig. 4). The results of the blood tests showed that the white cell and lymphopenia were normal or reduced [72]. The typical ICU admission chest CT images were bilateral, multiple lobular, and sub-segment consolidation areas [73]. Non-ICU patients demonstrated bilateral ground-glass opacity and sub-segmental consolidation areas by representative chest CT conclusions [74]. Laboratory studies found that the most frequent symptoms are cough (67.7%), and fever (87.9%) while diarrhoea is uncommon.. 82.1% of ICU admitted patients reported lymphopenia [75].

## Epidemiology

The exact degree of pandemic morbidity and death from 1918 is not known because influenza does not differ from other respiratory diseases without laboratory confirmation [76]. Autopsy samples analyzed are mostly the pulmonary tissue of the fatalities that died in the autumn of 1918 [77]. There are also missing epidemiological details. The flu was not a reportable disease or illness monitored before the pandemic in any provincial or federal public health organization. After the pandemic death became apparent in the autumn of 1918, In addition, communities began to have physical offices to register cases of influenza [78].

**Fig. 5:**
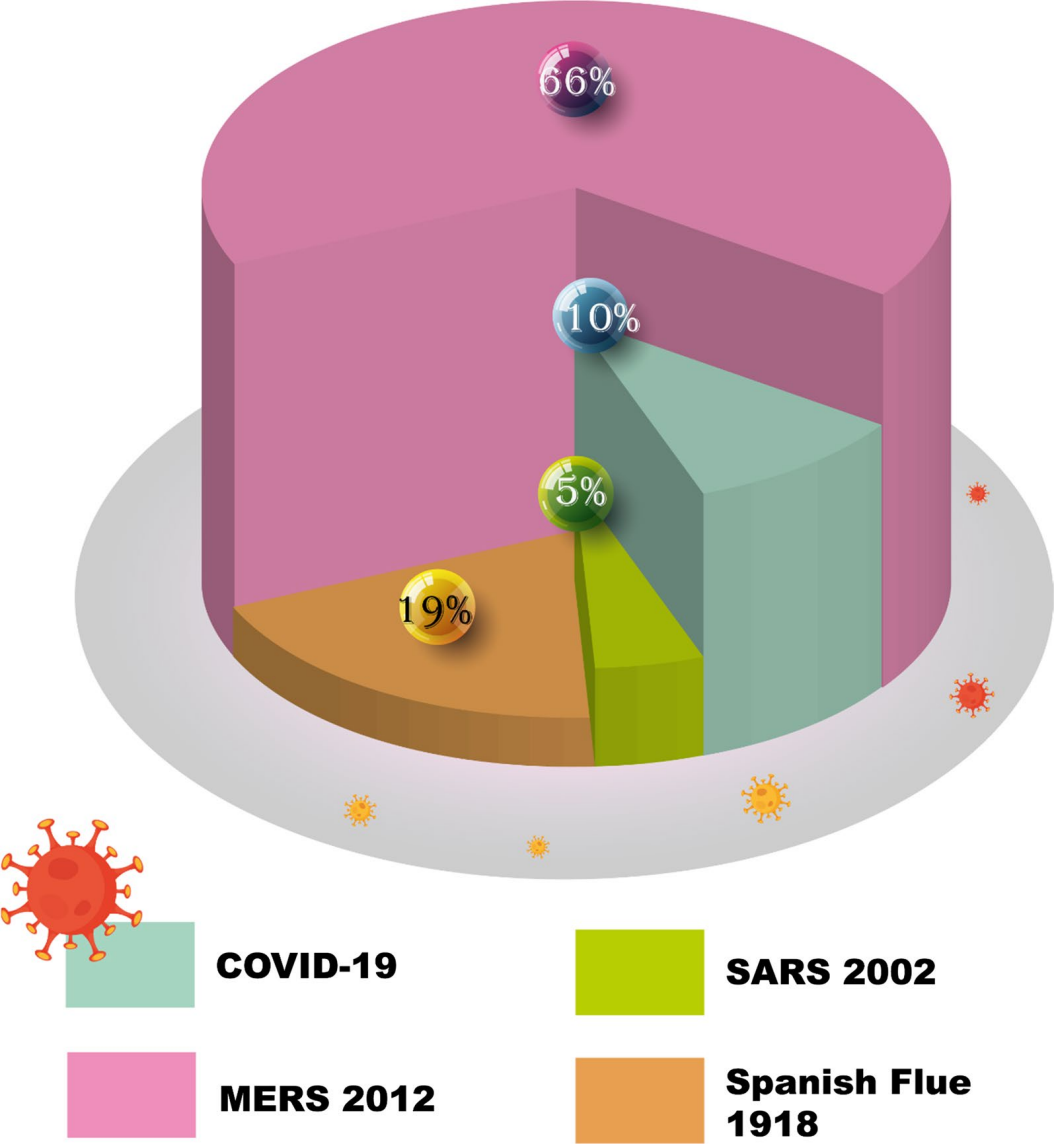
Case fatality rates (CFR) of different epidemics/pandemics

Nevertheless, numerous cases avoided reliable reporting and/or timely reporting. In the American Public Health Journal of January 1919, the editor wrote in several cases that data was incomplete and deceptive because "the demand for intervention was so strong, that very few were willing to focus on research in the future [60]. Reported since the middle of the eighteenth century, significant pandemics have occurred between 10 and 40 years. Of these, the pandemic of "Spanish flu" in 1918 was the worst in the world, killing 20–40 million or more people (Fig. 5) [79].

In 2002 a SARS outbreak originating in Guangdong in China triggered the number-one major infectious disease of the twenty-first century, with 916 deaths among over 8098 patients in 29 countries. Ten years later, 2254 laboratory reported cases of MERS-CoV were declared by the WHO, with 800 deaths in 27 countries, from 2012 to 16 September 2018 [80] (Fig. 5). Significantly, over eighty percent of recent studies in virology and genetics of this infection have demonstrated that both MERS-CoV and SARS bats could be potential natural reservoirs. of confirmed cases of SARS, 22 percent were health workers in China and more than 40 percent were health workers in Canada [81]. Similarly, MERS nosocomial transmission was in the Middle East and in Korea. The cases reported in the Middle-East and North Africa have all contributed to the outbreaks in other countries, and their transmission due to international travel. Both SARS and MERS contributed to massive public health and economic outbreaks [80].

## Host factors related to pandemic morbidity and mortality variations

### Immune responses and immunopathology

Dendritic cells (DCs) significantly contribute to innate immunity and can initiate large amounts of chemokines and cytokines [82]. These cells can move to the lymphoid tissue from peripheral tissue to activate the populations of T-cells [83]. On the other side, the key to immunity against viral infections is the adaptive T cells [84]; CD4 + T cells facilitate the virus-specific antibody production by T-dependent activation of B cells [85]. “CD8 + T cells” are nevertheless cytotoxic, killing viruses [86].

Inborn immune responses at the time of influenza can be defined as interactive between mucosal secretions and virions, epithelial-cell infections, and the activation of other types of residents of the epithelium or sub-epithelial layers [87]. Which involve lymphoid "innate" cells, resident macrophages, and dendritic cells (including alveolar macrophages) [87]. After the blood is recruited to the infection sites, other cell typologies like poly-morphonuclear leukocytes and monocytes take action [88]. Each cell type shows a different set of offers, they can sensitize the virus presence and enable special protective functions.

Besides, the receptors are situated strategically in several subcellular components like cell membranes, endosomes, cytosol, and mitochondria [89]. This allows the host to mount defences tailored to the invading pathogen and take its tropism, intracellular lifestyle, and reproduction strategy into account [90]. For flu and other infections, therefore, innate responses may be conveniently divided into modules, each of which involves specific cell types, receptors, molecules of the effectors, and intracellular compartments. The components of the influenza module enclose (1) solvable extracellular proteins containing corporal fluids; (2) the interferon system; (3) different kinds of cytokines and chemokines able to orchestrate an innate response; (4) macrophage and neutrophils phagocytosis; (5) dendritic cell antigen presentation [91].

In the case of SARS infection, As the innate and acquired immune responses help the control of viruses and mild diseases, cytokine dysregulation, viral cytopathic symptoms, ACE 2 lung downregulation, irregular immune response and autoimmune processes lead to a more serious illness and eventual death, the progression of SARS may be linked to cell-mediated immunity from T-helper (Th1) and inflammatory hyper-innate response [92].

Significant enhancement in"Th1 and inflammatory cytokines (interferon-g [IFN-g], interleukin-1 [IL-1], IL-6, and IL-12), along with a significant increase in chemokines like Th1 chemokine, IFN-g, IL-10 inducible, (IP-10) neutrophil chemokine, and monocyte protein-1 chemokine attractions were observed over two weeks after the onset of the disease in a research study conducted in 20 adults infected with SARS-CoV [18].

The immune response mechanisms caused by MERS-CoV infection and immune evasion strategies have not been fully explored yet. Of particular interest, MERS-CoV evolved innate immunity control strategies and prevented or blocked the pathways of IFN production [93]. This skill can substantively be responsible for the high death rate levels of MERS-CoV patients, particularly those with immune-compromised. Once the virus has been recognized as Toll-like Receptors (TLRs), one of the two different adapter molecules is recruited either MyD88 (Myeloid Difference Primary Response 88) or Toll / Interleukin-1 Receptor-(TIR-) domain-containing Adapter-Inducing Interferon-β (TRIF). The molecules also activate the MAPK and NF-ŚB pathways which promote the development of pro-inflammatory retardants and IFNs"[93].

During COVID-19 infection both inborn and adaptive immune cells are synergistically involved in the anti-viral response. The rate of lymphocytes and subsets of T cells that act a significant role in regulating the immune response differs with potential viral pathological mechanisms depending on the type of virus. A considerable rise in the neutrophils, leukocytes and neutrophil–lymphocyte ratio (NLR) was seen in severe cases of COVID-19 compared to mild cases [96]. Prominent lymphopenia, which indicates an impairment of the immune system, occurs in most patients with COVI-19, especially in severe ones [97]. Therefore, it appears that leukocytes and neutrophils may strengthen the cytokine storm (CS) other than the COVID-19 lymphocytes.

**Fig. 6:**
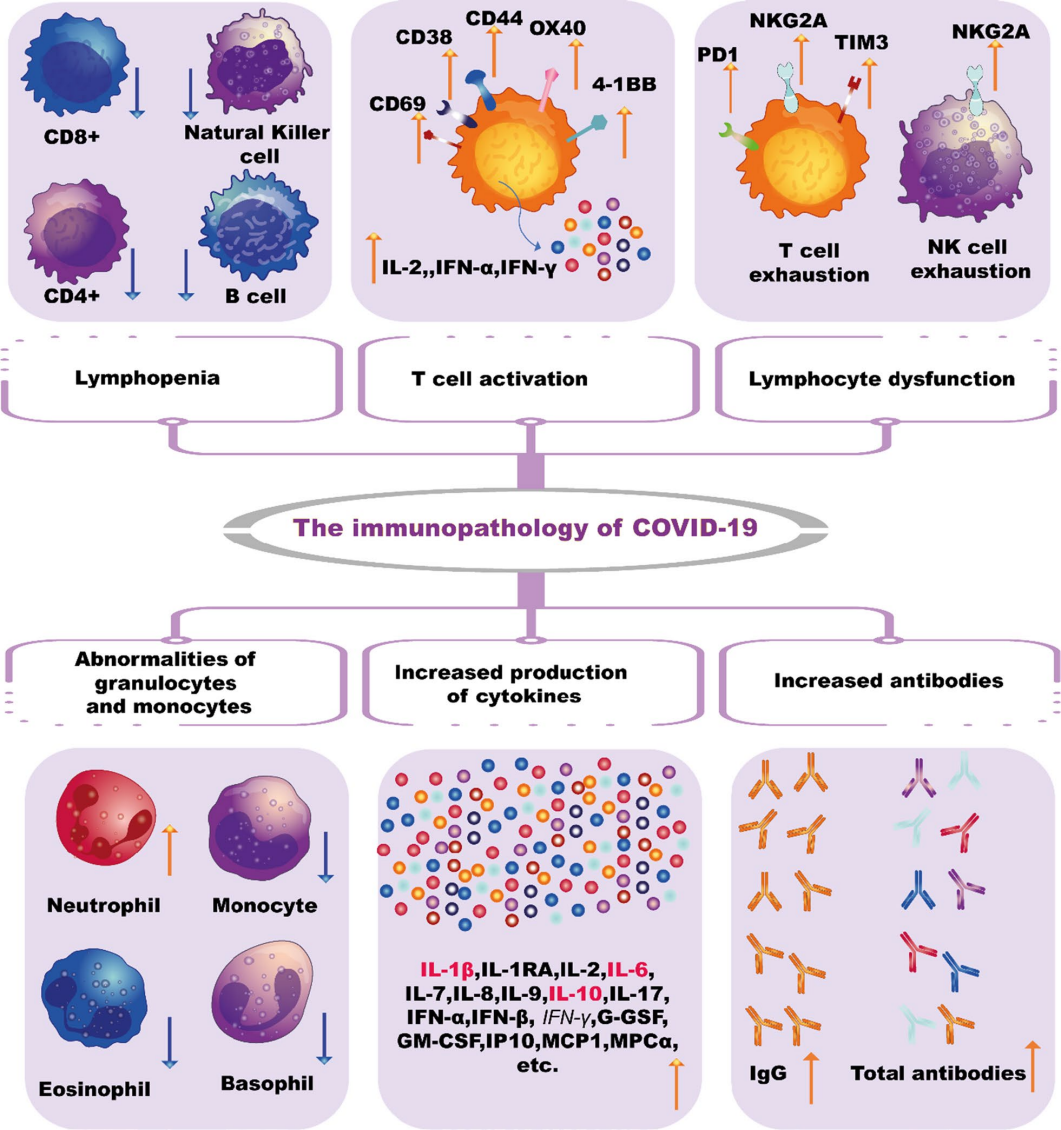
The immunopathology of COVID-19

Past work has decreased the overall number of lymphocytes and T cells in patients with SARS-CoV infection [94]. The infection by SARS-CoV-2 may result in immune disorders dysregulation by affecting the T cell subsets [95–97]. Significant T cell alleviation is observed in COVID-19 and is more pronounced in severe cases. In COVID-19 patients, the levels’ cells (CD3+, CD8+), cytotoxic suppressor and helper T cells (CD4+, CD3+) and regulatory T cells are lower than normal levels. In contrast, helper T cells and regulatory T cells are remarkably lower in severe patients than in non-severe patients as shown in Fig. 6 [98]. Regulatory T cells are known to be responsible for maintaining immune homeostasis by suppressing the activation, proliferation, and pro-inflammatory function of most lymphocytes, including NK cells, T cells CD+4, T cells CD+8, and B cells (Fig. 6) [99]. Also, the percentage of naive helper T cells amplifies.

In contrast, the percentage of memory helper T cells and CD28 + cytotoxic suppressor T cells decreases in severe COVID-19 cells [100]. The balance between the naive T cells and the memory T cells is fundamental to the effective immune response. Besides T cells, reduction of NK cells and B cells is observed in COVID-19 [101]. Overall, these results indicate that SARS-CoV-2 indicate that SARS-CoV-2 is responsible for immune misregulation with the induction of aberrant cytokine and chemokine responses [102], alteration of the lymphocyte subgroup, all of which could lead to cytokine storms and further tissue damage [103]. Excessive inflammatory response with a characteristic of cytokine storms cause serious illness and worsens the COVID-19 prognosis [104].

#### Age

The age of an affected individual play a main role in identifying their risk of deceased during the 1918 influenza pandemic. In general, when seasonal influenza death rates are plotted as a function the age of the population, a "U" formed curve is obtained, with the highest level of mortality observed among the young and the elderly [6]. Conversely, pandemic epidemics (to varying degrees) are characterized by a shift in lethality towards younger age groups. This was particularly marked during the 1918 pandemic when young adults (15–30 years) generally had a high mortality rate that a "W" mortality curve was generated [6].

For SARS, Individuals of all ages were infected, and the median age was less than Forty-five years. Healthcare workers represented 22% of all cases in Hong Kong and 22.8% in Guangdong. In Canada and Singapore, the percentage of healthcare workers affected was higher, at 43% and 41%, respectively. The age and gender distribution of SARS in Hong Kong is as follows: 61.7% of patients are under 45 years of age, 21.2% are between 45 and 64 years of age, and the remainder are over 64 years of age. Eleven (8.14%) of the 135 early community cases with no history of contact with SARS patients were zoonotic type. The lethality rate in Hong Kong rises with age as in other world regions: 14.7% among people under 44, 21.4% between 45 and 64 and 63.9% over 64. Experience from Hong Kong and other areas suggests that deaths are linked to co-existing diseases in the oldest age group (> 64 years) [105].

In MERS, older adults and people with health conditions such as diabetes, chronic lung disease, kidney disease, or cancer. Additionally, patients with weakened immune systems, such as those receiving chemotherapy or immunosuppressant medication, and most of those who died of MERS had pre-existing chronic diseases [68].

According to the center for disease control and prevention (CDC), COVID-19 is a new disease with limited information on risk factors. Little information is available on the risk factors for serious illness. Based on clinical expertise and currently available pieces of information, adults and individuals of any age who have severe underlying medical conditions might be at higher risk for severe illness from COVID-19 [106]. So far, we know that people at high risk of severe disease from COVID-19 are 65 years and older and who lives in a nursing home or long-term care facility. Individuals of all ages with underlying medical conditions, mainly if not well controlled, including:

1. People with severe to moderate chronic lung disease or asthma [107].
2. People who have serious cardiovascular problems [108].
3. People who are immune-compromised [109].
4. Numerous factors can make a person immunocompromised, including cancer treatment, smoking, bone marrow or organ transplants, immune deficiencies, poorly controlled HIV or AIDS, and prolonged use of corticosteroids and other immune-suppressing drugs. [110].
5. People with severe obesity (body mass index [BMI] of 40 or higher) [111].
6. People with diabetes [112].
7. People with the chronic renal disease under dialysis [113].
8. People with liver disease [114].

### Effect of pandemics on pregnancy

**Fig. 7:**
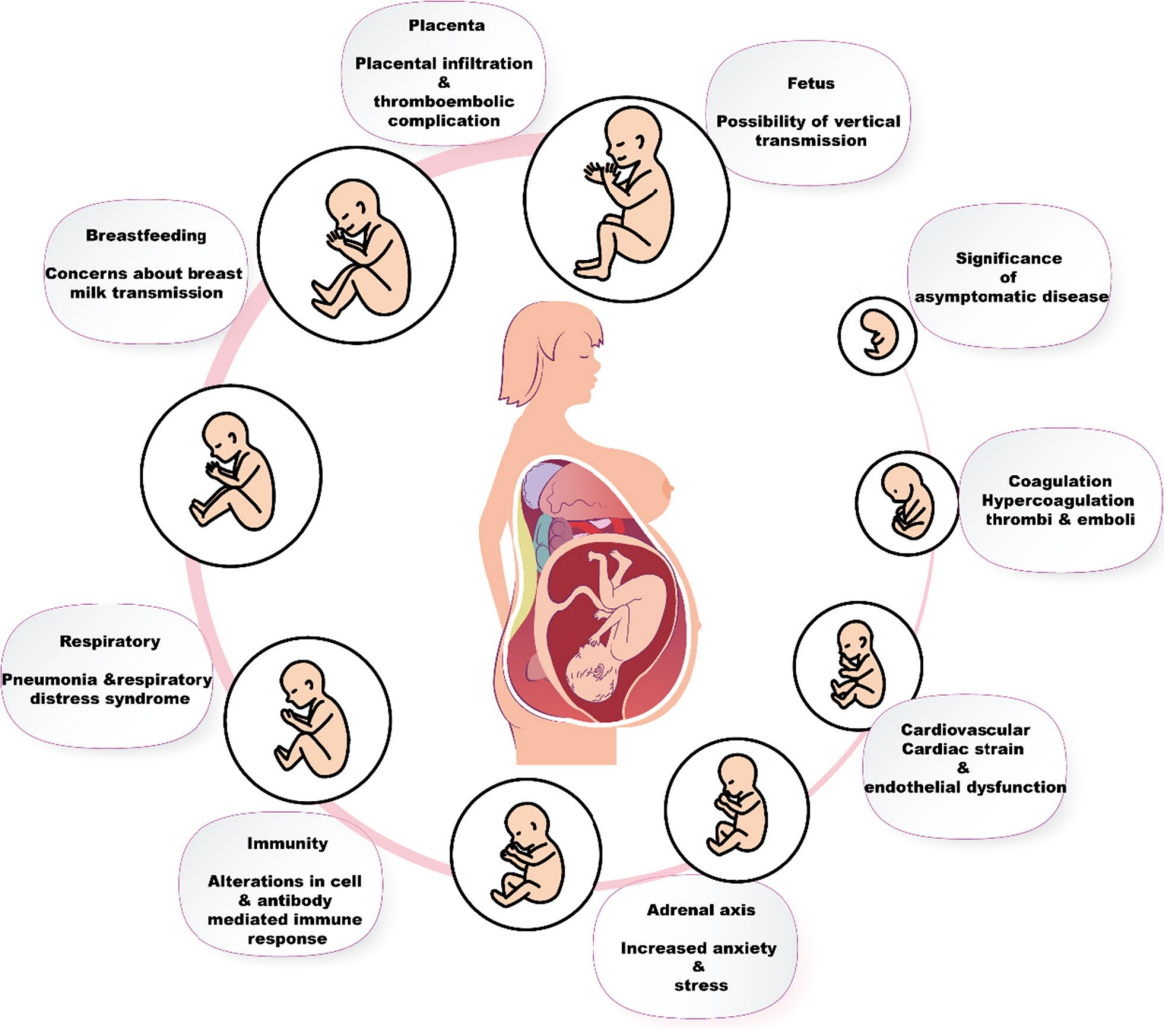
Effect of pandemics on pregnancy

Pregnancy is a source of risk for disease and death [115]. This is associated with several physiological various transformations that take place during pregnancy. Due to the hormonal and mechanistic changes during pregnancy, several changes also happen in respiratory and cardiovascular systems, particularly increased stroke volume, heart rate, reduced O_2_ consumption, and decreased lung capacity (Fig. 7) [116]. Immunologically relevant changes also occur during pregnancy, shifting from cell-mediated to humoral immunity [117]. Such changes can make pregnant women more vulnerable or more likely to be seriously exposed to specific viral infections, such as influenza [118]. While suitable Control groups that are not pregnant are usually not as available, the death rates for pregnant women in the 1918 and 1957 pandemics appear to be abnormally high. Of the 1,350 influenza cases reported in pregnant women during the 1918 pandemic, the proportion of deaths was 27% [119]. Likewise, 45% of pregnant women hospitalized with influenza died in 1918, and 20% of pregnancy-related deaths during the 1957 pandemic; half of the deaths of women of reproductive age were due to pregnancy [120].

Additionally, in spring 1919, birth rates in all populations examined declined by an average of 2.2 births per 1,000 people, representing a 5–15% decline from baseline levels [120]. The low birth rate in 1919 reached its lowest point 6.1–6.8 months after the flu pandemic peak in the autumn, indicating that the birth shortfall was due to an excess of first trimester abortions among ten expectant mothers during the pandemic peak. Pandemic-related mortality was not sufficient to explain the observed results [121].

SARS coronavirus infection has been a contributing factor to severe maternal illness, maternal death and spontaneous miscarriage [122]. In a case–control investigation to determine the effects of SARS on pregnancy, ten pregnant and forty non-pregnant women with the infection were compared. In terms of symptoms, renal failure and disseminated intravascular coagulopathy were more common in pregnant women with SARS than in those without SARS. As a result, 60% of pregnant patients with SARS requested admission to an intensive care unit (ICU), compared with 18% of the non-pregnant group [122].

Another study reported by Maxwell et al*.* [123], based on a clinical study of a group of seven pregnant women infected with SARS-CoV, showed a mortality rate of 28%, compared with 10% for the non-pregnant positive control group.

As a precautionary measure, SARS infected mothers have been recommended not to breast-feed their newborn children to prevent viral vertical transmission [122]. Most pregnant women displayed primary symptoms such as cough, dyspnoea and fever. The other main symptoms of COVID-19 in pregnant women are summarized in Fig. 7 [124–126].

## Burdens of COVID-19

The pandemic of COVID-19 has swept into over 200 countries with notable confirmed cases and deaths. It has caused mental health stress and public panic [127]. The rapid dissemination of COVID-19, which started in China, has been characterized as a pandemic by WHO in March 2020 [128]. In February 2020, Egypt announced its first COVID-19 case. After that, Egypt scaled-up precautionary measures, with a partial lockout beginning in March 2020 [129]. Reverse transcriptase-polymerase chain reaction (RT-PCR) was performed on nasopharyngeal swabs taken from symptomatic patients, and contacts of confirmed cases traced over the previous two weeks [130]. In individuals with a high suspicion rate, the test was repeated after 48 h [130]. The airport testing included body temperature and clinical assessment and the use of a rapid diagnostic test for severe-acute-respiratory syndrome IgM and IgG for coronavirus 2 [129].

The general public in China has severe anxious behaviors, resulting in a significant shortage of medical supplies. In addition, many frontline medical staff have been overworked for an extended period, which has prevented them from getting sufficient rest. There are indications that mental health problems may be occurring among health workers and survivors during the SARS epidemic [131]. However, the time distribution of the spread of COVID-19 may also commit to the heterogeneity of the disease burden across the US. The size and the time of the epidemiological peak, in particular, determine the required health system's responsiveness to provide proper healthcare. In many cases, it is challenging to obtain accurate data forecasts of the epidemic peak due to limited and often not trustworthy incidence data and the difficulties of modelling the effects of rapidly implemented and modified mitigation efforts [132]. Variability in county-level screening standards and actions, non-pharmaceutical interventions (NPIs) such as social distancing and outbreak initiation, and insufficient laboratory testing data also limit efforts to accurately model epidemic trajectories over several weeks [132]. In addition, projections of the cumulative burden of disease are less constrained by these difficulties. They do not attempt to describe the evolution of an epidemic over time. Even though these projections do not consider the nuance of the intensity and timing of outbreaks, their estimates of the disease burden's spatial footprint contain essential information for resource allocation [132].

## Non-pharmaceutical interventions

During viral pandemics, various strategies have been used to limit the virus's propagation and treat infected and affected patients, such as quarantine, mass gatherings, facemasks, and hygiene [133].

### Quarantine

For centuries, quarantine has been used to limit the emergence, introduction, transmission and spread of transmissible diseases [134]. When the “second wave” of Flu has been transformed into severe in 1918, Many nations have enforced stringent quarantine measures on all incoming carriers to prevent the propagation of the flu [6]. In most cases, these attempts failed. Restrictions have been put in place very late. The new viral infection was already well established in the country. The quarantine has been invoked by those infected who have not yet shown symptoms [135]. Thus, countries like the U K and S.A dismissed marine quarantine as inoperative and inefficient [6]. However, the Australian government imposed a naval quarantine before any victims of the 2nd wave were reported [136]. It helped protect Australia from the second pandemic wave until December 1918, when the quarantine was eventually finally over. The sea restrictions thus protected Australia from the pandemic. It indirectly helped protect some Pacific islands that were reliant on Australian supply boats. [137].

The most striking example is the difference in mortality between Western Samoa and American. A rigorous naval quarantine was enforced on American Samoa by the American Governor in 1918. This quarantine limited the flu from accessing the country, and no mortality cases from the 1918 flu were documented in American Samoa. This was in stark contrast to neighboring Western Samoa (∼ 100 km away), which did not have a strict sea quarantine. [6, 138]. Therefore, Western Samoa was infected by Tulane's New Zealand supply ship. Influenza is estimated to have claimed the lives of more than a quarter of the population [6].

In the case of SARS, prevention measures include early detection of cases and isolation, contact tracing and follow-up. Quarantine is efficient, but it is costly in time and resources and socially intrusive, so few countries can maintain such efforts over long periods [105]. Most quarantined individuals were confined to their homes during the SARS outbreak and active monitoring of symptoms [139].

In some countries, quarantine has been legally imposed and monitored by neighborhood support groups, the police, other workers, or home video cameras [139]. In other areas, consistency has been "requested", but judicial orders were handed down for a small percentage of non-compliant persons. According to reports, SARS was diagnosed in 0.2% of quarantined contacts in China-Taiwan, 3% in China-Hong Kong Special Administrative Region (SAR) and 4–6% in China-Beijing [139]. This is in part a result of different criteria for quarantining people. The most at-risk contacts (except for health care workers who have been exposed to certain unsafe conditions of patient care) was exposed to sick family members. Quarantine resulted in economic and psycho-social stress, risk communication, compensation and staffing issues for individuals, families, employers and governments [140]. Legitimate appeals and non-compliance with quarantine orders were rare [141].

Appropriate quarantine actions and measures that should be considered for MERS patients to prevent the spread of MERS-CoV to other susceptible individuals. Despite the fact that several studies in SK and SA have indicated that human-to-human transmission is relatively limited, it has been proven to be of very important, particularly in hospital outbreaks.[142, 143]. Accordingly, on 30 January 2020, Accordingly, WHO has determined that the coronavirus (COVID-19) disease is a public health emergency of international concern. As the epidemic continues to evolve, Member States are examining options to prevent the introduction of the disease into new areas or reduce human-to-human transmission in areas where the virus causing COVID-19 is already circulating [144].

Policy actions to reach these objectives may include quarantine, which implies restricting or separating from the rest of the population. These healthy people may have been exposed to the virus to monitor their symptoms and ensure early screening of cases. Many countries have the necessary legal authority to enforce quarantine [145].

**Fig. 8:**
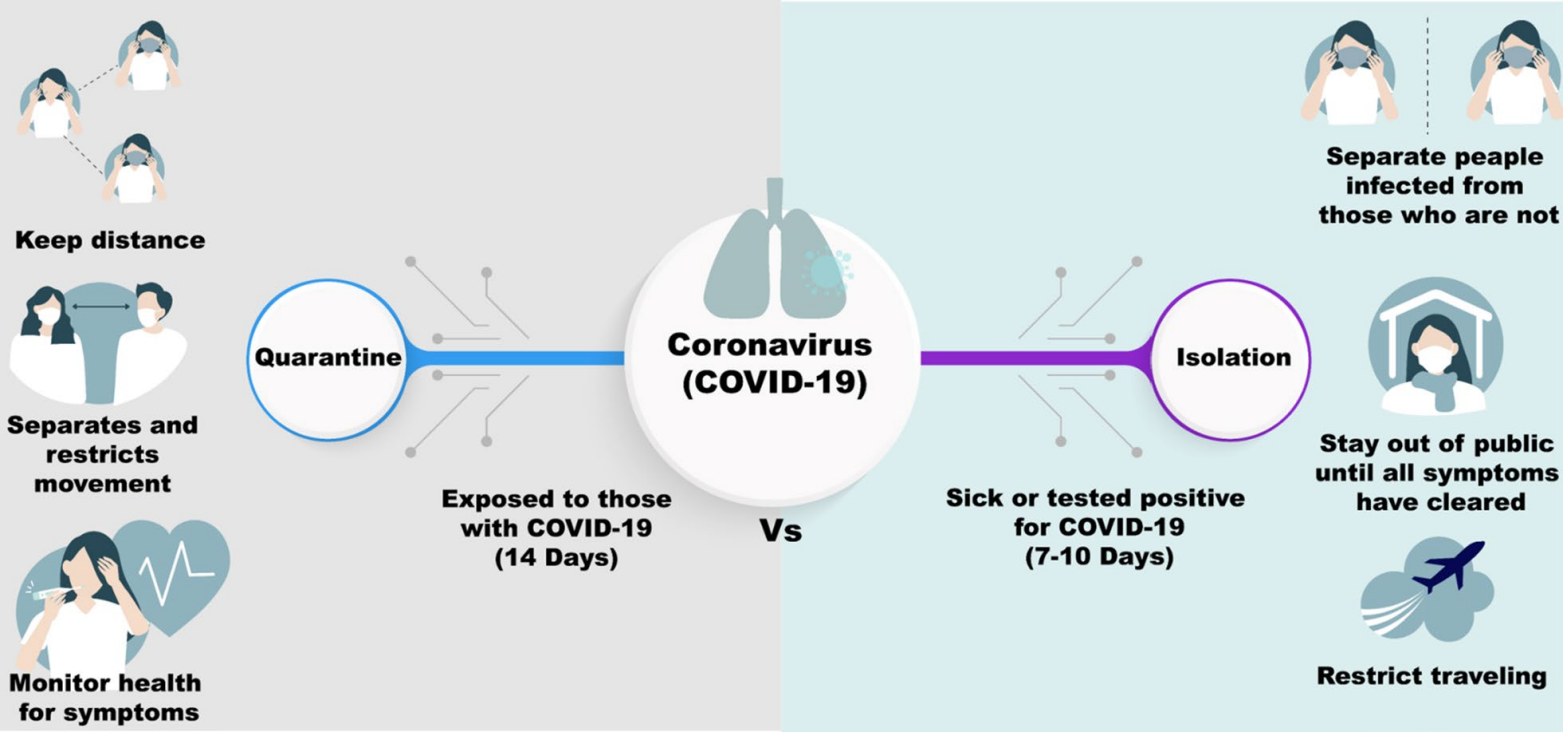
The difference between quarantine and isolation in COVID-19 pandemic

The WHO advises that contacts of patients with COVID-19 confirmed in tests should be quarantined for Fourteen days after the last exposure to the patient. To apply quarantine, a contact with a person who is involved in any of the following activities from 48 h before until 14 days after the onset of symptoms in the patient (Fig. 8) [144]:

- Having direct contact with a COVID-19 patient from within one meter and for > fifteen minutes;
- Providing direct care to patients with COVID-19 without the use of appropriate personal protective equipment;
- Staying in the same nearby environment as a COVID-19 patient (including sharing a workplace, classroom or home or attending the same gathering) for a while;
- Travelling nearby (i.e. within one meter) to a COVID-19 patient, regardless of the means of transport used.
- Other situations, as indicated by local risk assessments. [144].

As a point of comparison, no quarantine has been issued in the United States for the recent SARS-CoV or MERS-CoV outbreaks. The last United States federal quarantine was imposed in 1963 to prevent or avoid the spread of smallpox from Sweden to the United States during a Swedish smallpox epidemic [3].

### Mass gatherings, facemasks, and hygiene

The pandemic of 1918 involved many cities implementing simple interventions to limit the propagation of the disease. In particular, restrictions were placed on many community gatherings where human-to-human transmission might occur [146]. As a consequence, schools, theatres, universities and dances have been closed. At the same time, mass gatherings, such as marriages and funerals, are banned to avoid overcrowding [6]. The peak mortality rate was lower in cities that rapidly implemented these non-pharmaceutical interventions within days of the first local cases than those that waited a few weeks to respond [147]. Overall mortality was also affected by the timing of the lifting of these interventions. For example, while restrictions on gathering people helped reduce transmission of the influenza virus, actual viral transmission resumed after those restrictions have been reduced (usually within 2–8 weeks of their implementation) [148].

Facial masks were a standard preventive measure used during the 1918 pandemic [149]. Although the infectious agent of pandemic influenza was not particular, the consensus was that it was an airborne disease and that wearing a face mask would prevent infection [150]. Consequently, many cities and regions, including Guatemala City, San Francisco and some prefectures in Japan, have made it mandatory to wear a medical proper face mask in public places, and special teams and education campaigns have been set up to enforce this regulation [6]. However, for a facemask to be at least marginally effective against the influenza virus, it must be:

- Used at all times,
- Correctly made and fitted,
- Composed of proper material [151].

SARS social distancing measures were implemented to protect from the fast-spreading of the virus, such as closing schools, cancelling mass gatherings, theatres, public facilities, public transport, restaurants or hospitals. Face masks in areas of suspected widespread unrelated community transmission of SARS coronavirus has been applied. [152]. Many people in these areas also opted to wear masks outside their homes. These measures were often implemented simultaneously with other actions, such as increased contact tracing, making their independent effectiveness challenging to assess. However, the simultaneous introduction of various criteria has been associated over time with a dramatic decrease in the number of new SARS cases [139]. A study in Beijing demonstrate that using a mask more frequently in public places may be associated with increased protection. Another case–control study in China and Hong Kong found that wearing a mask frequently in collective places, wash your hands more than ten times a day and thoroughly disinfecting living quarters appeared to offer protection [153]. Except for the group in Amoy Gardens, where accidentally produced sewage aerosols transmitted SARS-CoV, the transmission of SARS in the community from aerosols or in social settings appears to be uncommon [154].

Notwithstanding the multiple mass gatherings that provided millions of opportunities for the virus to spread, no outbreaks of MERS or MERS-CoV were reported during or immediately after these events. [155]. According to Hui et al*.,* [156], some procedures used to enhance MERS control:

- Hand hygiene, droplet and contamination precautions for febrile patients before testing for MERS-CoV.
- Provide surgical masks to all patients undergoing haemodialysis and ensure that healthcare workers wear N95 filtering masks when caring for a patient with a confirmed MERS-CoV infection who is undergoing an aerosol-generating procedure.
- Patients suspected of being infected with MERS-CoV and admitted to dialysis or intensive care units should be placed in isolation rooms with a portable dialysis machine.
- Strengthen environmental cleaning and prevent non-essential staff and visitors from coming into contact with MERS-CoV infected patients.

Regarding COVID-19, since March 2020, cancellations of international and national religious, sporting and musical events have increased as countries around the world take steps to avoid or minimize the spread of SARS-CoV-2 [157].

Numerous high-profile GMs have been cancelled or rescheduled, including sporting events like the Union of European Football Associations Euro 2020 football championship, the Formula One Grand Prix in China, the Six Nations rugby championship in Italy and Ireland, the Olympic boxing qualifiers, the Mobile World Congress in Barcelona, and the Umrah in KSA [157].

Actions to limit the propagation of COVID-19 must be prioritized based on their expected multiple on effective R divided by their cost. By this criterion, experimentation with and deployment of universal masks looks highly desirable. Facial masks help reduce community transmission when used with widespread screening, contact tracing, quarantine of potentially infected persons, hand washing, and physical distancing. Through their effect on R0, all of these measures have the ability to minimize the period of containment needed. As governments talk about relaxing lockdowns, maintaining transmissions at a low enough level will be essential to preserve health care capacity until a possible vaccine can be produced. Masks may be essential to preventing a “second wave of infections” from over-burdening the health care system. Further studies and scientific research are urgently needed to overcome this emergency.

UNESCO declares that "where human activities may result in morally unacceptable harm that is scientifically plausible but uncertain, measures must be taken to avoid or mitigate such harm". This is known as the precautionary principle. The World Charter for Nature, adopted by the UN General Assembly in 1982, was the first international endorsement of the principle of prevention. It was implemented in an international agreement, called the Montreal Protocol of 1987. The Charter declares that the deaths and economic collapse that have already occurred as a result of COVID-19 are a morally unacceptable harm [158].

## Medical interventions: antiviral therapies and vaccines

### Antiviral therapy and other potential treatments

Despite spectacular advancements in medication drug therapy over the past decades, the causative agent of the 1918 influenza pandemic has been a mystery [159]. Without clear information on the agent responsible for the pandemic, a broad range of different therapeutic and preventive treatments have been inclines [160]. Individuals have experimented with drugs (including Aspirin) and home remedies such as mustard poultice, tobacco, beef tea, quinine, opium, saltwater, zinc sulphate inhalation, and alcohol [161]. As with the Japanese medicine Kampo (herbal remedies with green tea), the traditional Chinese medicine may provide a beneficial effect on the stimulation of perspiration (helping to lower fever), replacing lost fluids and improving vitamin C levels. Equally, when using traditional Chinese medicine may have reduced the illness's severity of the flu infections in some persons [6, 162]. Therefore, scientific studies are needed for the validation and determination of the active substances in order to be able to manufacture medicines based on these active substances on a large scale in the near future. Additionally, the dose-toxicity effect of these natural compounds needs to be seriously studied to prevent any potential side effects. Today, antiviral drugs are key factors in preventing and treating infection with the so-called influenza virus disease [163, 164]. In a regular influenza season, antiviral drugs are primarily which is used to cure or treat seriously ill patients, especially those with a weak immune system [164]. In the case of a pandemic, particularly in the period prior to a vaccination becoming available, anti-viral drugs are crucial to treat persons who have been infected and prevent infection among those who have been disclosed [165]. There are currently two drugs licensed for use against influenza, including adamantanes and NA inhibitors. Both rimantadine and amantadine are oral drugs that trigger the M2 ion channel of influenza A [166, 167]. The clinical use of these drugs is, unfortunately, is no longer recommended worldwide Because of the fact that the broad-based resistance of circulating influenza A viruses [167].

In particular, Inhibitors of NA target the enzymatic activity of the viral NA-protein [168]. Oseltamivir is administered as an oral oseltamivir phosphate, which is further transformed into its active carboxylate form in the liver [169]; Zanamivir is inhaled as a powder (which limits its use in people suffering from underlying respiratory problems) [170], and peramivir is administered intravenously, which is essential for persons who have been hospitalized [171]. The latter three drugs have been approved in the US, Australia, Europe, Canada, Japan, Korea, and Taiwan. They work by simulating sialic acid binding in the NA active site of A and B influenza viruses [169–171].

Both Zanamivir and Oseltamivir are efficient for prevention and post-exposure prophylaxis in persons [172]. However, these drugs have been randomized controlled trials in patients with less complicated influenza.; therefore, observational data should be used to monitor and evaluate the efficacy in critically ill and hospitalized patients, where the need is likely to be greater. Notwithstanding this limited scope, the results of the studies are systematically show improved outcomes from the use of NA inhibitors, including reductions in the incidence of pneumonia and hospitalization, and a reduction in the risk of hospitalized mortality. An additional consistency is that better results are obtained with early administration of NA inhibitors (within two days of the occurrence of symptoms). However, later administration may be of further benefit in critical situations [31]. The effectiveness of antiviral agents including ribavirin, INF, and protease inhibitors that was used to cure individuals with SARS-CoV infection in 2003 [173]. None of these therapies have proven benefit owing to a lack of prospective randomized, placebo-controlled clinical trial data. Supportive care continues to be the mainstay of treatment of SARS-CoV infection [173].

In the form of intravenous pulse methylprednisolone (MP), systemic corticosteroids were given to some patients with SARS-CoV infection for several reasons [174]. First, it was hypothesized that the clinical progression of pneumonia and respiratory failure associated with the peak viral load of SARS-CoV may be mediated by the host inflammatory response [175]. Treatment with systemic corticosteroid blocking agents considerably reduced MCP-1, IL-8, and IP-10 levels 5–8 days after treatment in twenty adults with SARS-CoV infection.[176]. In patients with fatal SARS-CoV disease, haemophagocytosis in the lungs was found, assigned to cytokine dysregulation. A therapy with systemic corticosteroids was therefore performed to modulate these immune responses [177]. However, prolonged use of systemic corticosteroid therapy may increase the risk of nosocomial infections, such as disseminated mycoses, metabolic derangements, psychosis, and osteonecrosis [178].

Recovering plasma, donated mainly by health care workers who had recovered entirely from SARS-CoV infection, appeared to be clinically helpful to the care other subjects with viral progression of SARS-CoV infection [179]. Delivery of convalescent plasma at an early stage appears to be more efficient and effective as, among eighty people who were infected with SARS-CoV received convalescent plasma at PWH, the discharge rate at day 22 was 58.3% for patients (n 5 48) treated within 14 days of onset of illness compared with 15.6% for those (n 5 32) treated beyond 14 days. In the absence of proven effective antiviral therapy, convalescent plasma and human monoclonal antibodies merit further investigation for the management of SARS-CoV if it returns [18].

Regarding MERS, various treatments already in existence and in development can be helpful antiviral such as ribavirin and mycophenolic acid (MPA) [180]. Currently, no specific treatments to treat ribavirin were empirically employed for serious of severe patients of MERS. However, there is no objective fact that they improve treatment performance [178]. Treatment with either lopinavir/ritonavir or IFN-b1b in the marmoset model was combined with better clinical, radiological and pathological results with lower viral loads compared to no treatment. In contrast, mycophenolic acid on its increases viral load and death rate [181].

In KSA, macrolide treatment usually begins before the patient arrives in intensive care [182]. In a study of 136 patients in a retrospective study, MERS patients, noted that macrolide treatment was not connected with reducing mortality or improvement in MERS-CoV RNA clearance [183]. A randomized controlled trial is underway in KSA. Comparative analysis of lopinavir/ritonavir, recombinant IFN-b1b, and standard supportive care against placebo and routine supportive care in patients with laboratory-confirmed MERS requiring hospital admission [184]. It was demonstrated that systemic corticosteroids delay viral clearance in critically ill patients with MERS-CoV infection.[185]. A range of anti–MERS-CoV drugs and host-directed therapies are considered potential therapies for MERS-CoV [183].

Antiviral and supportive treatments are clearly essential in treating patients with COVID-19 [186]. Because CS is frequently present in severe cases and is often the cause of the exacerbation, anti-inflammatory treatment can help prevent further aggravation [186]. As is well known, there are a number of types of anti-inflammatory medications, including non-steroidal anti-inflammatory drugs, chloroquine/hydroxychloroquine, immunosuppressant's, glucocorticoids, and inflammatory cytokines antagonists (such as TNF inhibitors, IL-6R monoclonal antibodies, IL-1 antagonists, Janus kinase inhibitors) [186, 187]. The use of corticosteroids may be justified in concert with the help of cytokine inhibitors such as anakinra (IL-1 receptor antagonist) or tocilizumab (IL-6 inhibitor). Intravenous immune globulin (IVIG) may also play a role in modulating an immune system that is in a hyper-inflammatory state [186]. Overall, the prognosis and recovery from this critical stage of illness are poor, and prompt recognition and application of such therapy may have the most significant yield [186, 188]. Table 1 displayed different approaches for the treatments against COVID-19 with the reaction mechanism.

**Table 1:** Different approaches used for the treatments against COVID-19 with the reaction mechanism

Drug	Mechanism of action	References
Remdesivir	Acts as an inhibitor of RNA-dependent RNA polymerase of coronaviruses	[16]
Favipiravir	An inhibitor of the RNA-dependent RNA polymerase (RdRp) enzyme, it acts as a purine nucleotide and inhibits viral protein synthesis. And recently, some studies have demonstrated its ability to induce lethal mutagenesis in vitro against SARS-CoV-2	[189]
Ribavirin	Acts as a guanosine analogue that ensures chain termination by inhibiting RNA polymerase and therefore limiting viral replication	[189]
Chloroquine (CQ) and hydroxychloroquine (HCQ)	CQ and HCQ are regulators of the immune system by affecting cell signaling and the expression of pro-inflammatory cytokines	[190]
Glucocorticoids	Glucocorticoids were utilized to reduce CS symptoms in patients with severe COVID-19 problems, including ARDS, acute kidney difficulties, acute cardiac injuries, and elevated D-dimer levels	[186]
Teicoplanin and other glycol-peptides	Act by inhibiting cathepsin B and cathepsin L in target cells	[189]
Monoclonal or polyclonal antibodies	Antibodies, both monoclonal and polyclonal can be proposed as prophylactic tools by targeting haemagglutinin binding against viral infections. Ongoing studies to develop monoclonal or polyclonal antibodies to the coronavirus are mainly targeting MERS-CoV2	[189]
Convalescent plasma	Convalescent plasma has been extensively recommended for COVID-19, but the effect of convalescent plasma cannot be discerned from the impact of the patient's concomitant diseases, stage of disease or impact of other treatments. Further investigations are desired to test and validate the efficacy of Convalescent plasma for the treatment of COVID-19	[191]
Herbal medicine	During the COVID-19 epidemic in China, some traditional Chinese medicines were widely used, such as *Astragali Radix, Glycyr-rhizome Radix Et Rhizoma, and Fructus forsythia*. Therefore, rigorous clinical trials on large populations should be conducted for the validation and determination of the active substances in order to be able to manufacture drugs based on these active substances on a large scale in the near future	[189]

### Vaccine

Vaccinating is one of the world's most successful ways to prevent disease, as indicated by the WHO. [192].”A vaccine helps the body’s immune system recognize and fight pathogens like viruses or bacteria, which then keeps recognize and fight pathogens like viruses or bacteria, which then recognize and fights pathogens such as viruses or bacteria, which protect us from the diseases they cause “[193]. Vaccinations protect from more than twenty five debilitating or life-threatening diseases, including polio, measles, tetanus, diphtheria meningitis, flu, typhoid and cervical cancer [194]. Nowadays, most children receive their immunizations on time. However, nearly twenty million people worldwide still miss outputting them at risk of serious diseases, death, disability, and ill-health [195].

First inactivated influenza vaccine was mono-valent (influenza A) [196]. In 1942, a bi-valent vaccine was produced after discovering the influenza B virus. It was later identified that the influenza viruses mutate, leading to antigenic changes [197]. WHO has published annual recommendations since 1973 for the influenza vaccine composition based on the results of systems of surveillance that identify currently circulating strains [198]. In 1978, the first trivalent vaccine included two strains of influenza A and one strain of influenza B. Currently, two strains of influenza B are circulating; the most recent WHO guidance propose adding a second B strain to make a quadrivalent vaccine [197].

Moreover, currently available inactivated seasonal influenza vaccines may even prevent the induction of cross-reactive CD8 + T-cell responses, which are our primary protection in a pandemic. They may therefore prove to be a double-edged sword [199]. Prompt production of vaccines also remains a challenge for future influenza pandemics. This was particularly evident during the 2009 pandemic, when sufficient quantities of pandemic vaccine were not available until October 2009, well after the pandemic had spread worldwide [200]. Vaccine production can be even more complicated because certain avian influenza viruses can cause the death of embryonated chicken eggs needed for vaccine production..[201]. Different vaccine strategies are required to accelerate vaccine production and overcome these problems. Nevertheless, An influenza vaccine that provides broad-spectrum, long-lasting immunity remains the gold standard for pandemic planning [6]. Continued research is needed to understand how a universal influenza vaccine can be implemented.

In SARS, S protein ensures an essential function in the regulation of the viral infection through binding receptors and membrane fusion between the virus and the target cell [202]. An adenovirus-vaccine-based can stimulate potent SARS-CoV-specific immune responses in rhesus macaques and is promising for the establishment of a vaccine to combat SARS-CoV [203]. Other researchers have pointed out that the gene S DNA vaccine can induce the expression of specific IgG antibodies to SARS-CoV effectively in mice, with a seroconversion rate of 75%.after three immunization doses. In contrast, virus replication was decreased by over six orders of magnitude in the respiratory tracts of mice injected with S-plasmid DNA expression vectors. Protection was provided by a so-called humoral immune mechanism [204, 205]. The recombinant S protein showed antigenicity and receptor binding capacity. In contrast, synthetic peptides that elicit specific antibodies to the S-CoV S protein could be an alternative approach to SARS vaccine development [202, 206].

There is currently no vaccine that can protect against MERS-CoV infection. Many research groups are working on developing a using various platforms and several strategies, and some have shown their effectiveness in animal models [183].

Vaccination is perhaps the preferred choice for controlling COVID-19 [207, 208]. Epitopes, mRNA, and S protein-RBD structure-based vaccines have been widely proposed and started [209]. Rapid reconstruction of SARS-CoV-2 using a synthetic genomics platform has been reported, and this technical advance is helpful for vaccine development. [210]. The human ACE2 and rhesus monkey transgenic mouse models of COVID-19 have been well established for vaccine development [211]. A number of SARS-CoV-2 vaccines are already in ongoing clinical trials [205].

### The relation between the Bacillus Calmette–Guérin (BCG) vaccine and COVID-19

Tuberculosis vaccine Bacillus Calmette-Guérin (BCG) is a lively attenuated vaccine developed at the beginning of the twentieth century at the Pasteur Institute in Paris [212, 213]. Since that time, it has been the most widely used vaccine. Globally, with approximately one hundred and thirty million children being vaccinated each year [214]. However, it is interesting to note that shortly after its first introduction in Europe in the nineteen-twenties, epidemiological studies indicated that BCG vaccination greatly reduced infant death rate [212].

More recently, BCG vaccination has been shown to be correlated with reduced case death rate for COVID-19. The latest data from publicly available resources also indicate that the incidence of COVID-19 and the total number of deaths are strongly associated with the presence or absence of national mandatory BCG vaccination programmers [215].

On the basis of clinical results and experimental data, it is assumed that BCG induces long-lasting immune system changes that lead to enhanced responses to infections in both innate and adaptive immunity. [216]. In innate immune cells, BCG induced histone modifications and epigenetic reprogramming at the promoter sites of genes coding for inflammatory cytokines such as interleukin (IL)-1, IL-6 and tumor necrosis factor (TNF). This process has been termed "trained immunity" [217].

"In two studies, BCG was evaluated in Japan and BCG in Denmark for inducing cytokine secretion in peripheral blood lymphocytes"[218]."One study, carried out in Africa, demonstrated that BCG Japan caused more robust proliferation of CD4 + and CD8 + T cells, higher secretion of Th1 (interferon-c, TNF-a and IL-2) and lower secretion of Th2 cytokines (IL-4) compared to BCG Denmark [218]. Another study in Mexico showed that "BCG Japan induced higher levels of IL-1a, IL-1b, IL-24 and IL-6 in peripheral blood mononuclear cells obtained from vaccinated children, compared to BCG Denmark"[219]."These results suggest that BCG Japan is more effective than BCG Denmark in inducing the production of several types of inflammatory cytokines"[215].

## Nanotechnology between COVID-19 diagnosis and immune system boosting

Intravenous immunoglobulin (IVIg) therapy decreases intestinal epithelial cell infectivity. It reduces the growth of the opportunistic *Candida albicans* (human unicellular-fungal pathogen) in the murine gut in connection with the upregulation of anti-inflammatory cytokines coupled with downregulation of proinflammatory mediators [220].

CoVs cover positive-stranded-RNA viruses relating to the Coronaviridae family [221]. Viral RNA genome sequencing has shown that the virus-producing COVID-19 is phylogenetically linked to the SARS-associated CoV initial separated in Chinese horseshoe bats through 2015–2017. CoVs are extremely deadly with human-to-human communication, which has grievously-produced several losses. Unluckily, we were collectively disappointed to know that the opportunity was apparent, imminent, and important. We declined to ensure that the policies and directives, produced by specialists, can be quickly and efficiently performed in case of a disorder [222, 223].

### Nanoparticles (NPs) and immune system

Intravenously introduced nanomaterial of natural and inorganic sources is growing attention in clinical sciences [224]. Macrophages (a kind of immune cell covering cellular waste, bacteria, and other unknown particulate materials) of the spleen and the liver immediately prevent blood-borne bits. This is doubtful if the preferred target for healing NMs is the preferred target remains outside human organs [225].

Still, the adsorbed and functionalized blood protein on the outsides of NMs, but not the polymer layer, accidentally and predominantly induced activation of the other pathways [226]. On cover adsorption, plasma proteins may support conformational and thermodynamic differences and consequently, display reactive combinations that may convert responsive to C3b initiative. Ultimately, this makes C3bBb and C3bBbP convertases connected to cover-adsorbed protein. This method may be explained as a non-specific general mechanism by which NMs, despite their chemical structure and composition, could trigger the other pathway of the whole way. Moreover, it is stated that protein-C3b and protein-C3 convertases are produced and delivered from the NMs outside. [227]. As C3b is an opsonic fragment, connected C3b coupling and relief (in the frame of protein-C3b) may reveal why long-circulating NPs are gradually accepted and removed from the human blood by the macrophages of the spleen and liver.

### Antimicrobial NPs as antivirals

The antimicrobial characteristics of some NPs like Ag NPs are quite recognized [228]. The importance of various NPs is being FDA approved for complete wound closure. It is possible for the antimicrobial colloidal silver compounds, passed by breath, to reduce the inflammation of respiratory system diseases. It is proven that Ag NPs have antimicrobial and antiviral characteristics [229]. However, there is no accurate study about the possibilities of breath application of NPs for the restriction and/or processing of respiratory diseases. Therefore, before the safe use of Ag NPs, several steps should be conducted (a) defining optimal Ag NPs metal features for most maximum powerful anti-viral structure, (b) expected useful inhibitory concentration to be taken at the objective respiratory system area, and (c) the necessary dosage for effective implementation by breath control. To determine the necessary delivery dosage, an advanced system must be designed to assess the impact of all platforms among the aerosol stocks by the aerosolizing material to the ultimate tissue displacement. In the context of breaths, the Ag NPs solution is proper. The critical opening point for determining any adequate dosage is to set the necessary point inhibitory concentration of the active factor.

The standard anti-viral potential is achieved with NPs with less than 10 nm. In production, the NP's size stabilization (stabilizing or capping agents) process changes the anti-viral power. The current gum acacia capping mainly hinders the antiviral impact. Furthermore, it seems that Ag NPs of size less than 10 nm have more powerful anti-viral potential than Ag NPs with size more than 25 nm [230].

Communication among different NPs and the immune system becomes significant, and there are foundational issues regarding the security of the synthesized NPs. NPs can interact with many vital elements (cells, proteins, receptors, etc.) or activate cell signaling pathways, and subsequently, create variable immune replies (suppression and/or activation) and also severe medical conditions (cancer and/or autoimmune diseases) [231].

Directed NPs can be created to individually-associate with or withdraw identification by the immune system. Synthetic NPs have been used regularly to make new immunotherapy approaches. Immunotherapy includes the intentional infliction of the immune system as a therapeutic approach [232]. One of the main forces of immunotherapy is that there can be limited harmful side outcomes than those connected with conventional treatments [233].

### Nanoparticles (NPs) and immune cytokines

**Fig. 9:**
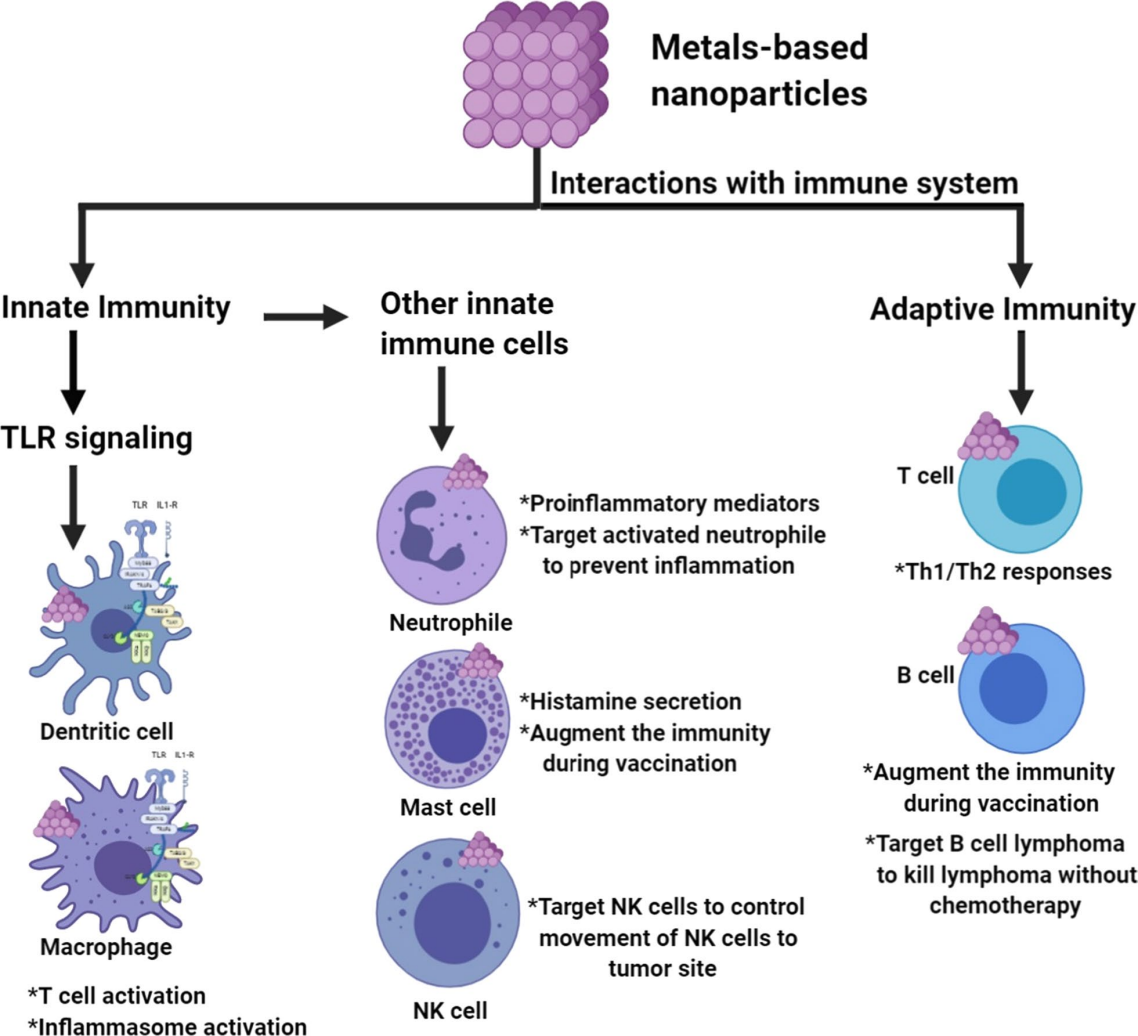
The role of metal-based nanoparticles for activating immune cytokines

The surface of NPs can be altered with special receptors to be attached with particular targets. In the innate immune system, phagocytosis relies on the stability of the signals of prophagocytic and antiphagocytic on target. For example, the copper oxide (CuO) NPs approach followed in the up-regulation of heat-connected proteins and stimulated ROS. Also, gold nanoparticles (Au NPs) caused up-regulation of the provocative mediator (NF-κB) that is negotiated within dysregulation of the immune homeostasis after stopping the function of the TIPE2 (tumor necrosis factor-α-induced protein 8-like 2) protein [234]. Proinflammatory cytokines may be caused by Toll-like receptors (TLR) indicating pathways. Several tumor necrosis factor and cytokines can stimulate inflammatory groups, improve the vascular permeability, and produce inflammation during severe inflammatory responses [235]. Cytokines are primary mediators of heat release (fever) [236]. TNF-α stimulates endothelial cells starting to hypotension. Some directed NPs can also stimulate inflammasome signaling pathways [237, 238]. Titanium dioxide (TiO_2_) NPs and crystalline silica (SiO_2_) NPs produce the inflammasome and cytokine discharge in bone marrow-acquired macrophages [237, 238] as displayed in Fig. 9.

Peeters et al*.* lately stated that SiO_2_ stimulated inflammasomes in the lung epithelial cells and basic bronchial epithelial cells, which increased the inflammatory signal and induced fibroblast generation. Ag NPs effected inflammasome development and triggered cytokine discharge [239].

Inflammasome-stimulation-related cytokine creation by dendritic cells in reply to particle treatment was size-subject. The activity was achieved when the synthesized NPs were between 500 and 900 nm. Yazdi et al. stated that SiO_2_ NPs and TiO_2_ NPs, stimulate the inflammasome, leading to cytokine discharge [240]. The NP's specificity for selecting and invading tissues in symptomatic imaging and drug-based treatments is essential to block non-specific cell junction in normal human tissue. The organization of the nano-formulations has tried to overcome this force by intense, magnetic targeting. Some NPs have been utilized for diagnostics, several usually Au NPs, and magnetic NMs. Au NPs for diagnostics DNA small sections may be connected to Au NPs with a 13.0 nm small diameter NPs. These connections over a sensor outside occur after a corresponding target. Au NPs are particularly-efficient designs for sensors due to various scientific methods that may be utilized to identify them [241].

Table 2 summarized nanomaterials that have been used for COVID-19 treatment, diagnosis and immune system boosting with their reaction mechanism.

**Table 2:** Representative nanomaterials (NMs) for COVID-19 treatment, immune system boosting and diagnosis

Types	NMs	Mechanism of action	References
NMs for COVID-19 Treatment	Polydopamine-poly (ethylene glycol) nanoparticulates	Encapsulates DNase-1 to degrade cell-free DNA, potently inhibits NETosis factors in blood samples from COVID-19 patients and also enhances survival in a sepsis model	[242]
	Nanorods coated with cell membrane	Prevent infection with hACE2 available on the cell membrane	[243]
	Thin shell polymer	Encapsulate catalase to degrade free radical oxygen	[244]
NMs for COVID-19 immune system boosting	Liposomes	Delivered a recombinant trimeric spike as a vaccine. It is a single-dose intranasal vaccination; it induces IgA production in the mucosa	[245]
	Self-assembled protein NPs with virus-like particle core	Display several distinct RBDs in vaccine form. The RBDs are linked to NPs via SpyTag: SpyCatcher; various RBDs induce cross-reactivity against different coronaviruses	[246]
	Saponin-based nanoemulsion named Matrix-M1	Supply adjuvant activity for a recombinant spike protein vaccine. The M1 matrix is a mixture of two saponin-based fractions to balance adjuvant and adverse effect activities	[247]
	Self-assembled protein NPs with ferritin core	Present recombinant RBD and/or heptad repeat (HR) subunits of spike on the surface of protein NPs as a vaccine. The spike is bound to the NPs via Spy Tag: Spy Catcher. HR can induce cross-reactivity with other coronaviruses	[248]
NMs for COVID-19 diagnosis	Colloids gold-NPs	Detection of antinuclear IgM in blood on a lateral flow strip. The test is rapid and takes only 15 min to be read with the naked eye	[249]
	Gold-NPs conjugated to hACE2	Detecting the viral particle in serum samples. The test is rapid and takes only 15 min to complete; read with a microplate reader or smartphone-connected device	[250]
	Gold-NPs conjugated to antisense oligonucleotides and a graphene layer	Detection of viral RNA in nasal swab or saliva samples. The test is rapid, with a 5-min incubation time after extraction of viral RNA; detection of the electrochemical signal on a biosensor chip	[251]
	Selenium NPs	Detection of anti-nucleoprotein IgG and IgM in blood on a lateral flow strip. Testing is rapid and takes 10 min; can be read with the naked eye	[252]
	Magnetic NPs functionalized with streptavidin	Detection of anti-spike IgG in blood via afiltration column. The reading is achieved by means of a portable magnetic reader	[253]
	Polymer NPs	Detection of viral RNA on a lateral flow strip after RT-LAMP amplification. The proposed test is readable with the naked eye	[254]
	Nanoflakes of reduced-graphene-oxide	Sensing anti-spike and anti-RBD antibodies on a 3D printed test chip. Anti-spike and anti-RBD antibodies were immobilized on the surface of the nanoflakes, which are connected to gold electrodes; the test chip provide its reusability easy regeneration and smart reading tool with a smartphone	[255]
	Cobalt-functionalized TiO_2_ nanotubes	Electrochemical biosensor detection of RBD antigen. The measured signal is based on rapid electrochemical detection in 30 s without the need of antibody immobilization	[256]
	Polystyrene-NPs	Screening for anti-nucleoprotein IgG in blood on a lateral flow test strip. Prompt test in ten minutes; using a portable fluorescence reader as detector	[257]

## Cytotoxicity of nanoparticles for in vivo application

Nanotoxicity is an evolving field used to assess the unintended hazardous effects of nanoparticles (NPs) on human health. While NPs have become promising tools for a wide range of biomedical applications, their extensive use depends on the assessment of their biosafety [245, 248, 258–261]. There is increasing interest in assessing the health impact of these materials and expanding knowledge of their cytotoxicity and biocompatibility. Once a new nanomaterial appears, its cytotoxic effect, i.e., the possible alteration of basic cellular functions, is usually evaluated primarily. Nevertheless, the absence of cytotoxicity does not confer to these materials an implicit biocompatibility [262].

This must be assessed as a separate endpoint. The concept of biocompatibility is based on the adequate interaction between the nanomaterial and its biological environment, i.e. the non-existent toxic or immune response of the treated biomaterial (cell, tissue or organism) [262]. Cytotoxicity is generally related to the possible negative impact on a specific cell line. Thus, cytotoxicity is generally assessed first by specific tests conducted in vitro before being assessed in vivo. These tests can be performed in vitro (metabolic activity, cell proliferation and viability, oxidative stress, apoptosis tests, necrosis tests, etc.) and in vivo (behavioral analysis and body weight, biodistribution, biodegradation and clearance, pharmacokinetics, hematology and serum chemistry, histopathology, acute and repeated dose toxicity, reproductive and developmental toxicity, genotoxicity and mutagenicity, etc.) tests [262].

Regardless of whether in vitro or in vivo methods are used, the results of studies conducted on the toxicity of NPs are currently contradictory. Basically, it has been observed that cytotoxicity and biocompatibility are governed by several factors, including the inherent physicochemical properties of nanoparticles and the way they are delivered to the body. For example, a higher toxicity of nanoparticles was observed during oral and intraperitoneal administration compared to intravenous injection. In addition, biocompatibility was highly tissue or organ specific. The cytotoxicity of nanoparticles is also strongly related to physicochemical characteristics such as size, shape, surface area, and charge. All these elements show that the biocompatibility of nanoparticles is highly dependent on several factors, ranging from the intrinsic properties of the particles to the formulation, the biological target, the dose and even the methodology used to assess their toxicity [263].

In general, the smaller the size of the nanoparticles, the greater the cytotoxic effect. One of the hypotheses that could explain the toxicity of small-sized nanoparticles is that it results from the presence of a high surface area compared to their volume. This leads to an increased absorption capacity and may increase the risks of interaction with biomolecules [262, 263]. In addition, the dose of NPs administered to a model organism can also affect their toxicity. Similarly, Coradeghini et al. [264] revealed that the toxicity of Au NPs towards mouse fibroblast cell lines was dose-dependent.

Recently, Donskyi et al.[265], proposed graphene with precise double sulfate/alkyl functionalities as a platform for the inhibition of SARS-CoV-2 and feline coronavirus replication by virtue of viral envelope disruption. Notwithstanding using a wide concentration window (10 to 100 times), the graphene platforms show strong antiviral activity against native SARS-CoV-2 without significant toxicity against human cells. Hence, more research is needed on the effective doses and likely toxic effects of NPs to create a safe environment for humans against extremely dangerous diseases like COVID-19. Therefore, new specific standardization and certification tests (which include evaluation of physicochemical characteristics, sterility, pyrogenicity, bio-distribution and ADME, pharmacokinetics, and in vivo and in vitro toxicity) for preclinical nanosafety and toxicity risk study need to be developed. Efforts to standardize risk assessment procedures for NPs are ongoing and need to be further improved. Currently, nanomaterials are considered in the same way as conventional chemicals. The main efforts to standardize nanotechnology are being developed by the standards development organizations (SDOs).

## Biodegradability of nanoparticles for in vivo application

**Fig. 10:**
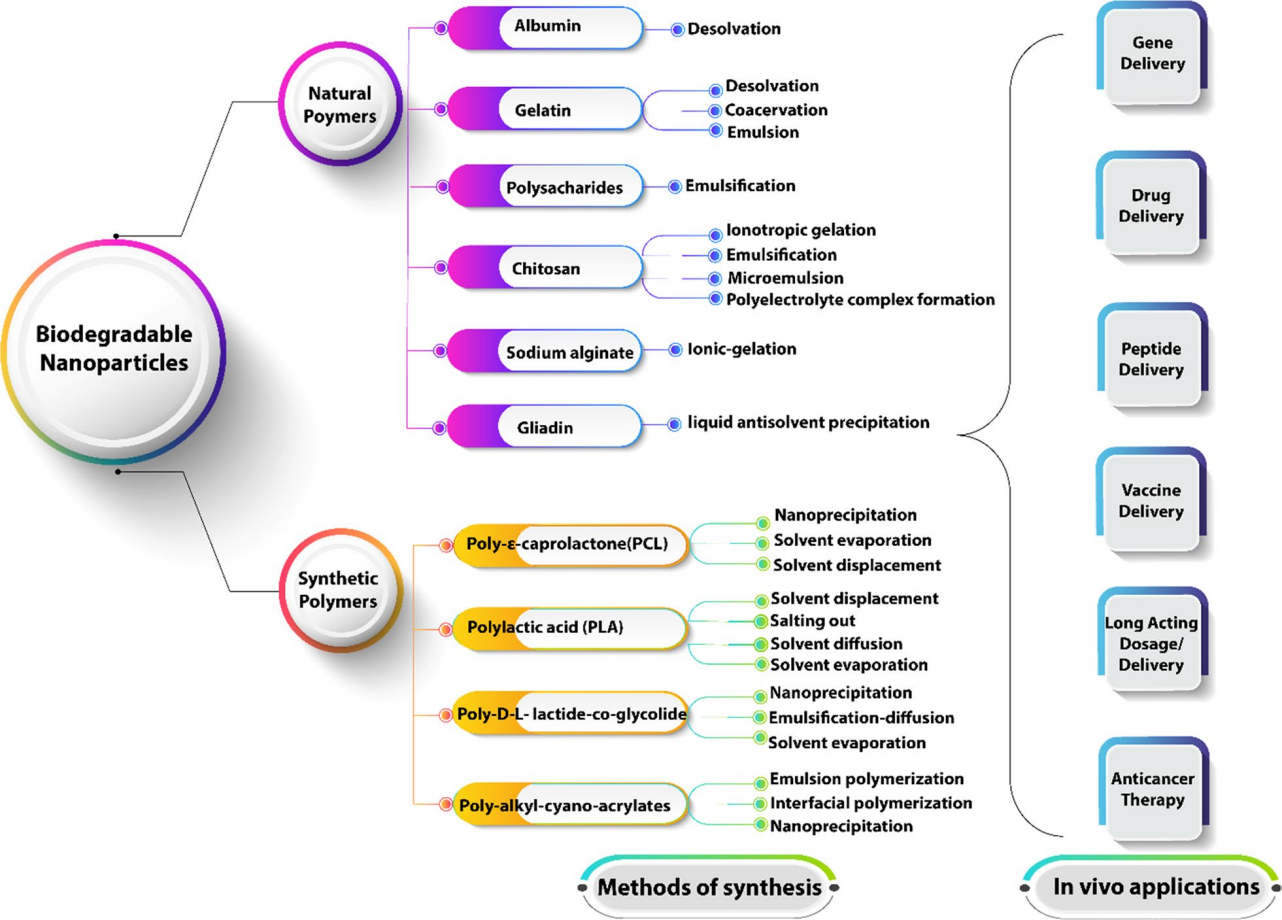
Synthetic pathways for biodegradable nanoparticles and their in vivo applications

Biodegradable nanoparticles (BNPs) are novel carriers for the delivery of drug molecules. They are receiving increasing attention due to their ability to serve as viable carriers for the specific delivery of vaccines, genes, drugs and other biomolecules to the body (Fig. 10). BNPs have become popular recently due to their special features such as targeted drug delivery, better bioavailability and therapeutic efficacy to deliver the drug at a constant rate. They offer improved biocompatibility, superior drug/vaccine encapsulation and convenient release profiles for a number of drugs, vaccines and biomolecules for use in a variety of applications in the field of nanomedicine [266–269].

Polymeric nanoparticles are considered as biodegradable material. They are polymeric colloidal elements of very small size in which a drug of interest can be encapsulated or incorporated into their polymeric network or conjugated or adsorbed onto the layer. Various natural and synthetic polymers are used in the synthesis of biodegradable nanoparticles (Fig. 10), some of the frequently used polymers are chitosan, cellulose, gelatin, gliadin, polylactic acid and polylactic-co-glycolic acid. Nanoparticles have been progressively explored for drug delivery and have enabled sustained kinetic release. Drugs integrated in this system can give better efficacy, decrease drug resistance, reduce systemic toxicity and symptoms, and also improve patient compliance [268].

Recently, Qiao et al. [270], proposed a peptide-based subunit candidate vaccine against SARS-CoV-2 delivered by biodegradable mesoporous silica nanoparticles induced high humoral and cellular immunity in mice. Through this study, seven linear B-cell epitopes and three CD8 + T-cell epitopes were selected from the SARS-CoV-2 spike glycoprotein by immune computational approaches for vaccine design. A nanoparticle-based candidate vaccine (B/T@BMSNs) against SARS-CoV-2 was promptly prepared by encapsulating these ten epitope peptides into BMSNs, accordingly. The BMSNs, endowed with potential biodegradability and excellent in vitro and in vivo safety, demonstrated the ability to efficiently deliver the epitope peptides into the cytoplasm of RAW264.7 cells. Strong humoral and cellular Th1-like immunity was induced by B/T@BMSNs in mice and the 10 selected epitopes were identified as effective antigenic epitopes capable of inducing a robust peptide-specific immune response [270].

To optimize NPs as a delivery system, a better understanding of the different mechanisms of biological interactions and particle engineering is still needed. However, biodegradable NPs seem to be a promising system for drug delivery due to their versatile formulation, sustained release properties, subcellular size, and biocompatibility with various tissues and cells of the body. The development of nanomedicine remains a great challenge and other biodegradable nanomaterials still need to be explored and validated for their potential clinical use. Figure 10 provides a summary of the synthetic routes for biodegradable nanoparticles and their in vivo applications.

## Conclusion and future perspectives

During the years 1919, 2002, 2012 and 2019, the world was attacked by four viral respiratory diseases, Spanish flu, SARS, MERS and COVID-19, respectively. Coronaviruses are single-stranded, non-segmented, enveloped, RNA-positive viruses that have a particular appearance under negative-stain electron microscopy. It is well known that wild waterfowl are the source of all influenza viruses in other species. Regarding COVID-19 fatigue and cough is myalgia or tiredness, most frequently reported symptoms. Sputum, headache, hemoptysis, and diarrhea were less frequent symptoms, and in over half the patients, dyspnea developed. During COVID-19 infection both inborn and adaptive immune cells are synergistically-involved in the anti-viral response. A significant increase in neutrophils, leukocytes, and neutrophil lymphocyte ratio has been identified in serious or critical cases of COVID-19 compared to mild cases. From currently available information and clinical expertise, the elderly and people of all ages with serious underlying health problems may be at increased risk of severe illness from COVID-19. The occurrence of pregnancy has been a strong risk factor for increased illness and mortality for both pandemic and seasonal influenza. During viral pandemics, a variety of different approaches were employed to limit or avoid the fast spread of the virus and treat infected patients such as quarantine, mass gatherings, facemasks, and hygiene. Today, anti-viral drugs are key in preventing and treating influenza virus infection and disease. Different medicines and approaches had been used for the treatments against COVID-19 such as remdesivir, favipiravir, ribavirin, chloroquine and hydroxychloroquine, glucocorticoids, teicoplanin and other glycol-peptides, monoclonal or polyclonal antibodies, convalescent plasma, and herbal medications. Vaccination is one of the most powerful ways for disease control and prevention, according to the WHO. A vaccine has the effect of helping the body's immune system to recognise and fight pathogens including the following viruses or bacteria, which then keepsrecognize and combat pathogens such as viruses or bacteria, which then recognize and fights pathogens like viruses or bacteria, thus protecting us from the diseases they induce. Intravenously introduced nanomaterial of natural and inorganic sources is growing attention in clinical sciences. The standard antiviral potential is achieved with NPs with less than 10 nm. In production, the NP's size stabilization (stabilizing or capping agents) process changes the anti-viral power. The NPs surface can be altered with special receptors to be attached with particular targets. In the innate immune system, phagocytosis guided by the stability of the signals of prophagocytic and antiphagocytic on target. This review presents insights about using NMs to give treatment to COVID-19 further, improve the bioavailability of the abused drugs, diminish their toxicity, and improve their performance.

## Data Availability

The datasets used and analyzed during the current study are available from the corresponding author on reasonable request.
